# N-acetyltransferase 10 promotes glioblastoma malignancy *via* mRNA stabilization of jumonji and AT-rich interaction domain containing 2

**DOI:** 10.1016/j.jbc.2025.108544

**Published:** 2025-04-25

**Authors:** Takuto Inoki, Akito Tsuruta, Yoshinori Masakado, Yuichiro Kai, Yuya Yoshida, Naoya Matsunaga, Shigehiro Ohdo, Satoru Koyanagi

**Affiliations:** 1 Department of Pharmaceutics, Faculty of Pharmaceutical Sciences, Kyushu University, Higashi-ku, Fukuoka, Japan; 2 Department of Clinical Pharmacokinetics, Faculty of Pharmaceutical Sciences Kyushu University, Higashi-ku, Fukuoka, Japan

**Keywords:** RNA acetylation, NAT10, cancer stem cells, glioblastoma, JARID2

## Abstract

Glioblastoma (GBM) is the most common and aggressive form of malignant brain cancer, with a poor prognosis and a 5-year survival rate of approximately 15%. The malignancy of GBM, including its treatment resistance and high recurrence rate, is largely attributed to the presence of cancer stem cells. Recent studies have identified the N-acetyltransferase 10 (NAT10), an enzyme responsible for catalyzing *N*_4_-acetylcytidine (ac4C) modification in RNA, as a key factor in cancer biology, with diverse roles across multiple cancer types. However, the specific contribution of this RNA modification to the malignancy of GBM remains unexplored. Here, we demonstrate that NAT10 expression is associated with poor prognosis in GBM patients and that NAT10 promotes GBM malignancy by enhancing stemness properties in human GBM cell line U251 and A172. A search for the underlying mechanism of NAT10-mediated enhancement of GBM stemness led to identification of polycomb repressive complex 2 (PRC2)-related genes as an epigenetic regulator. NAT10 mediates the acetylation of the coding region of Jumonji and AT-rich Interaction Domain containing 2 (JARID2) mRNA, which results in increased mRNA stability and elevated protein levels. Notably, the knockdown of JARID2 significantly reduced GBM stemness, suppressed tumor growth, and extended the survival of xenograft mice. Our findings suggest that NAT10-mediated acetylation of *JARID2* mRNA up-regulates its protein levels, thereby promoting stemness and contributing to the malignancy of GBM. Targeting this NAT10-JARID2 axis may represent a novel therapeutic approach for treatment of GBM.

Glioblastoma (GBM) originating from glial cells is the most common malignant brain cancer, classified as grade 4 by the World Health Organization (WHO). The current standard of care for GBM includes surgical resection, when feasible, followed by multidisciplinary treatment consisting of radiotherapy and chemotherapy with temozolomide, a second-generation alkylating agent (1). Conventional treatment for patients with GBM may extend survival; they do not result in a cure (2) largely due to the presence of GBM stem cells. Cancer stem cells possess self-renewal capacity, resistance to therapy, and high invasive potential, with residual cells leading to tumor recurrence. Therefore, eliminating GBM stem cells is crucial for achieving a definitive cure of GBM (3, 4). To achieve this, comprehensive analyses, including genome-wide association studies (GWAS) (5) and single-cell RNA sequencing analyses (6) have been conducted to identify the mechanisms that sustain GBM stem cells. Although these studies have revealed several underlying mechanisms, effective therapeutics remain elusive.

RNA modifications regulate physiological processes and disease states by modulating mRNA stability and translation efficiency (7), with over 150 types of RNA modifications having been identified (8, 9). These modifications have been closely linked to cancer progression, as exemplified by early mechanistic studies demonstrating that m5C modification in tRNAs is associated with the acquisition of aggressive properties in anaplastic thyroid cancer cells (10). Similarly, m5C modification to *GRB2* mRNA causes promotion of tumorigenesis and progression in squamous cell carcinoma (11).

A recent study has identified mRNA acetylation as a novel RNA modification in mammals (12). Acetylation of mRNA at the N4 site of cytidine (ac4C) by N-acetyltransferase 10 (NAT10) stabilizes mRNA and enhances translation efficiency. In human embryonic stem cells (hESCs), NAT10 expression is correlated with cellular pluripotency and the maintenance of self-renewal capacity through the acetylation of *POU5F1* mRNA (13). Furthermore, mRNA acetylation has been implicated in the malignancy of cancer cells, promoting cell proliferation and collagen synthesis in gastric cancer (14), as well as driving chemoresistance in bladder cancer (15). Although a negative correlation between NAT10 expression and prognosis has also been reported in GBM patients (16), the relationship between NAT10 activity and GBM stemness remains unknown.

Dysregulation of epigenetic modification has been implicated in cancer malignancy. In particular, trimethylation of histone H3 at lysine 27 (H3K27me3) has been associated with the development of various cancer phenotypes. In fact, H3K27 mutations, which result in the loss of H3K27me3, promote tumor development in diffuse intrinsic pontine glioma (17). The polycomb repressive complex 2 (PRC2) is the primary writer of H3K27me3 and consists of three core components: enhancer of zeste 1 and 2 (EZH1 and 2), embryonic ectoderm development (EED), and suppressor of zeste 12 homolog (SUZ12). PRC2 is recruited to its methylation sites by interacting with several other proteins, including metal response element binding transcription factor 2 (MTF2), jumonji and AT-rich interaction domain containing 2 (JARID2), AE binding protein 2 (AEBP2), and elongin BC and polycomb repressive complex 2 associated protein (EPOP) (18). Notably, JARID2 plays a crucial role in regulating the balance between self-renewal and differentiation in stem cells by the fine-tuning recruitment of PRC2 to chromatin and its subsequent modification (19, 20, 21). Although JARID2 has also been implicated in the malignant transformation of cancer cells, its role in GBM and its association with cancer stem cells remain unclear.

In this study, we demonstrated that the *NAT10* knockout attenuates the malignancy of U251 and A172 cells, a human GBM cell line, through repressing their stemness properties. RNA-seq analysis revealed that NAT10 stabilizes *JARID2* mRNA *via* ac4C modification, leading to up-regulation of JARID2 protein expression. Similarly, knockdown of JARID2 also attenuated the malignancy of U251 and A172 cells by suppressing GBM stemness. Our findings highlight the role of mRNA acetylation by NAT10 in maintaining GBM stemness through *JARID2* mRNA stabilization and suggest that targeting this mechanism may provide a novel approach for the treatment of GBM.

## Results

### Correlation of NAT10 expression with GBM malignancy

**Figure 1:**
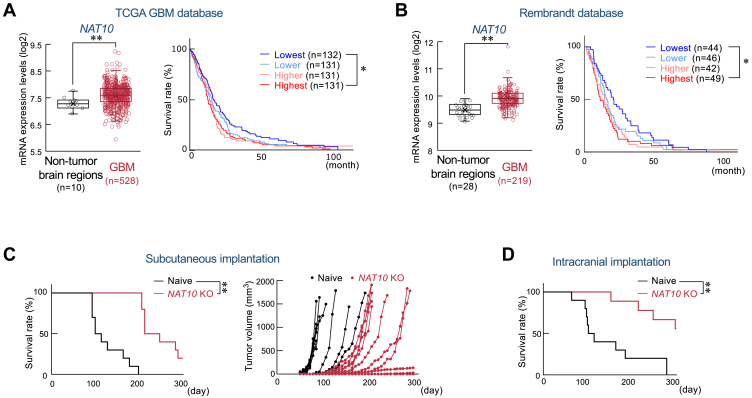
**Relationship between NAT10 expression levels and GBM malignancy**. *A and B*, the data from patients with glioblastoma (GBM) were obtained from the TCGA GBM database (HG-U133 A) (*A*) and the Rembrandt database (*B*) and analyzed using GlioVis. Dot plots in each panel show *NAT10* mRNA expression levels in GBM tissues and the non-tumor brain region. ∗∗; *p* < 0.01 significant difference between the two groups (Mann-Whitney *U* test). *Right* graphs show Kaplan-Meier survival curves for patients with GBM categorized based on *NAT10* mRNA expression levels: highest (>75%), higher (75∼50%), lower (50∼25%), lowest (25%>) based on *NAT10* mRNA expression levels. ∗; *p* < 0.05 significant difference between the two groups (Log-Rank Holm-Sidak test). *C*, the effect of *NAT10* knockout (KO) on the malignancy of U251 cells in the subcutaneous implantation model mice. Naive or *NAT10* KO U251 cells were subcutaneously implanted in Balb/c nude mice. The *left* graph shows Kaplan-Meier survival curves of naive or *NAT10* KO U251 tumor-bearing mice (n = 10). The *right* graph shows the tumor volume for each individual mouse. ∗∗; *p* < 0.01 significant difference between the two groups (Log-Rank Holm-Sidak test). *D*, the effect of *NAT10* KO on the malignancy of U251 cells in the orthotopic implantation model mice. Naive or *NAT10* KO U251 cells were intracranially implanted into Balb/c nude mice. The data show Kaplan-Meier survival curves of naive or *NAT10* KO U251 tumor-bearing mice (n = 10 for Naive, n = 9 for *NAT10* KO). ∗∗; *p* < 0.01 significant difference between the two groups (Log-Rank Holm-Sidak test).

To investigate the relationship between NAT10 expression and malignancy of GBM, we explored the mRNA levels of NAT10 in non-tumor brain regions and GBM tissues, as well as the survival time of patients with GBM, using GlioVis, data visualization tools for brain tumor datasets (22) (http://gliovis.bioinfo.cnio.es/). In the TCGA_GBM datasets, *NAT10* mRNA expression was significantly increased in GBM tissues compared to non-tumor brain regions (Fig. 1*A* left). Higher NAT10 expression in patients with GBM was associated with poorer prognosis (Fig. 1*A* right). Similar results were also obtained by analyzing the Rembrandt datasets (Fig. 1*B*). Given the relevance of NAT10 expression levels with prognosis in patients with GBM, we employed the human GBM cell line U251 cells to investigate whether NAT10 regulates GBM malignancy. To achieve this, we prepared *NAT10* knockout (KO) U251 cells using the CRISPR/Cas9 system and implanted them subcutaneously into the right back of Balb/c-nude mice. Mice-bearing *NAT10* KO U251 cells exhibited significantly prolonged survival and reduced tumor growth compared to those bearing naive U251 cells (Fig. 1*C*). Additionally, in an orthotopic xenograft model, *NAT10* KO also significantly extended survival (Fig. 1*D*). These data suggest that NAT10 plays a critical role in GBM development and prognosis.

### Dysfunction of NAT10 alleviates aggressive properties of GBM cells

**Figure 2:**
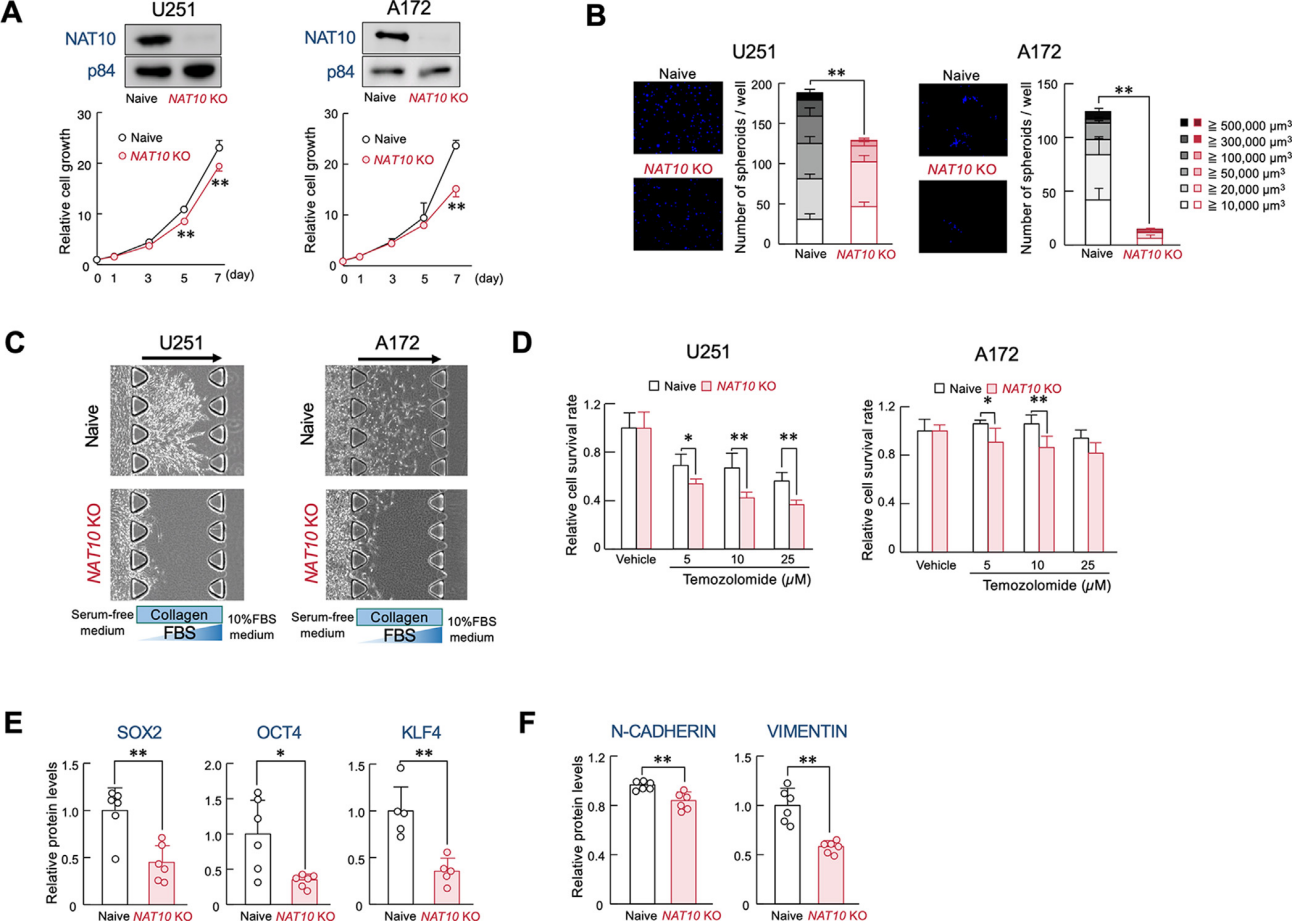
**Suppression of GBM stemness by knockout of *NAT10***. *A*, the proliferation of naive and *NAT10* knockout (KO) U251 and A172 cells. Values show the mean with S.D. (n = 12). The cell viability of seeding day (day 0) was set at 1.0. ∗∗; *p* < 0.01 significant difference from naive group at corresponding time points. (*F*_9,110_ = 69.073 *p* < 0.001 for U251 cells, *F*_9,110_ = 2331.346 *p* < 0.001 for A172 cells; ANOVA with the Tukey-Kramer *post hoc* test). Each *upper* panel shows the NAT10 protein levels in naive and *NAT10* KO U251 and A172 cells. *B*, the spheroid formation ability of naive and *NAT10* KO U251 and A172 cells. Each left panel shows a representative photograph of Hoechst33342-stained spheroids-formed by naive or *NAT10* KO U251 or A172 cells. Each *right* panel shows the number of spheroids and the distribution of their diameters. Values show the mean with S.D. (n = 5 for U251 cells, n = 4 for A172 cells). ∗∗; *p* < 0.01, significant difference between the two groups (*t* = 13.355 for U251 cells, *t* = 2.663 for A172 cells, Welch’s *t* test). *C*, the invasion ability of naive and *NAT10* KO U251 and A172 cells. Microphotographs show invasion of cells into 3D collagen gel. *D*, the sensitivity of naive and *NAT10* KO U251 and A172 cells to temozolomide. Naive and *NAT10* KO cells were treated with indicated concentration of temozolomide for 96 h. Values show the mean with S.D. (n = 8 for U251 cells, n = 6 for A172 cells). Cell viability of vehicle-treated groups was set at 1.0. ∗∗; *p* < 0.01, ∗; *p* < 0.05; significant difference between the two groups at corresponding concentration points. (*F*_7__,56_ = 55.596 *p* < 0.001 for U251 cells, *F*_7,40_ = 7.381 *p* < 0.001 for A172 cells; ANOVA with the Tukey-Kramer *post hoc* test). *E*, protein expression levels in SOX2, OCT4, KLF4 in naive or *NAT10* KO U251 cells. Protein levels were normalized to those of p84 expression levels. Values show the mean with S.D. (n = 5–6). ∗∗; *p* < 0.01, ∗; *p* < 0.05; significant difference between the two groups (*t*_10_ = 4.134 for SOX2, *t*_10_ = 3.001 for OCT4, *t*_8_ = 4.491 for KLF4, Student’s *t* test). *F*, protein expression levels in N-CADHERIN, VIMENTIN in naive and *NAT10* KO U251 cells. Protein levels were normalized to those of β-ACTIN expression levels. Values show the mean with S.D. (n = 6). ∗∗; *p* < 0.01 significant difference between the two groups (*t*_10_ = 3.979 for N-CADHERIN, *t*_10_ = 5.635 for VIMENTIN, Student’s *t* test).

To investigate how NAT10 promotes the malignancy of GBM, we investigated the proliferation and stemness properties of *NAT10* KO U251 and A172 cells. Although the growth ability of both *NAT10* KO U251 and *NAT10* KO A172 cells was slightly, but significantly, decreased compared to their respective naive cells (Fig. 2*A*). By contrast, spheroid formation ability, a hallmark of cancer stemness, was significantly decreased in both KO cells (Fig. 2*B*). GBM malignancy is also characterized by high invasiveness and chemoresistance (23). To assess the invasive potential of GBM cells, we performed a collagen-based invasion assay using a 3D cell culture chip. In both U251 and A172 cells, invasive potential was decreased by dysfunction of NAT10 (Fig. 2*C*). Dysfunction of NAT10 also enhanced cytotoxic effects of temozolomide (TMZ), a standard chemotherapeutic agent for GBM, on both cell lines (Fig. 2*D*). These data suggest that NAT10 contributes to the malignancy of GBM by promoting aggressive traits such as stemness, invasiveness, and chemoresistance. This notion was also supported by the observation that dysfunction of NAT10 decreases the protein expression of key stem cell regulatory factors—SOX2, OCT4, and KLF4 (24, 25)—as well as epithelial-mesenchymal transition (EMT) markers, N-CADHERIN and VIMENTIN (Fig. 2, *E* and *F*).

### NAT10 regulates GBM malignancy through regulating JARID2 expression

**Figure 3:**
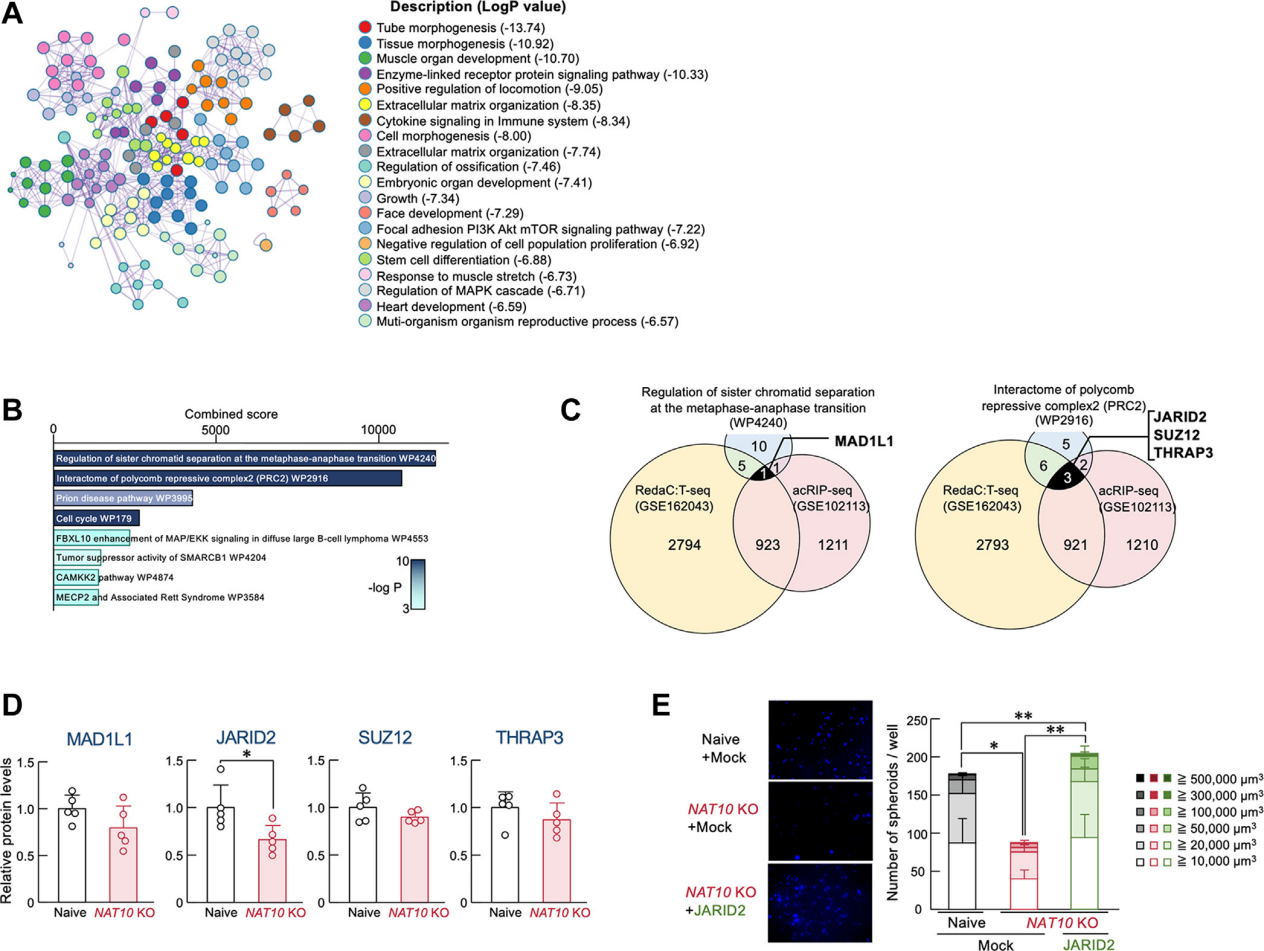
**NAT10 regulates the expression of JARID2 protein in U251 cells**. *A*, pathway and process enrichment analysis was performed using Metascape on 1217 genes that exhibited altered mRNA expression levels due to *NAT10* knockout (KO). Each term is represented by a circle node, where the size corresponds to the number of input genes associated with that term, and the color indicates its cluster identity. *B*, a schematic of diagram illustrates the search for NAT10-regulated genes responsible for stemness of U251 cells, utilizing analysis tools, ChIP-ATLAS and Enrichr. *Left* panel depicts schematic image of the gene search process. *Right* panel shows the results of Enrichr analysis using the Wiki_pathway_2021_Human dataset. *C*, identification of RNA-acetylated genes was performed based on three criteria: the enriched pathways shown in Fig.3B, acetylated genes identified from RedaC:T-seq (GSE162043) and acRIP-seq (GSE102113). *D*, protein expression levels of MAD1L1, JARID2, SUZ12, and THRAP3 in naive and *NAT10* KO U251 cells. Protein levels were normalized to those of p84 expression levels. Values show the mean with S.D. (n = 5). ∗; *p* < 0.05 significant difference between the two groups (*t*_8_ = 1.682 for MAD1L1, *t*_8_ = 2.686 for JARID2, *t*_8_ = 1.384 for SUZ12, *t*_8_ = 1.173 for THRAP3, Student’s *t* test). *E*, the spheroid formation ability of naive and *NAT10* KO U251 GBM cells. The *left* panel shows a representative photograph Hoechst33342-stained spheroids formed by Mock-transfected naive, *NAT10* KO, or JARID2-expressing *NAT10* KO U251 cells. The *right* panel shows the number of spheroids and the distribution of their diameters. Values show the mean with S.D. (n = 6). ∗∗; *p* < 0.01, ∗; *p* < 0.05 significant difference between the two groups (Kruskal-Wallis test with Mann-Whitney *U* test).

To assess the changes in the intracellular environment of *NAT10* KO U251 cells, pathway and process analysis was performed using the naive and *NAT10* KO U251 cells RNA-seq data (Table S1) by Metascape (26). A total of 393 genes were identified as showing greater than two-fold up- or down-regulation in *NAT10* KO U251 cells (Table S2). Enrichment analysis revealed that these gene sets were significantly associated with stemness- and differentiation-related terms, such as tissue morphogenesis, embryonic organ development, and stem cell differentiation (Fig. 3*A*). GBM stemness is maintained not only by the intracellular environment but also by the extracellular stimuli (4, 27). Therefore, we investigated whether extracellular factors are also involved in NAT10-mediated maintenance of GBM stemness. To this end, we performed a spheroid formation assay of naive and *NAT10* KO U251 cells under the same culture conditions. The EGFP-expressing naive U251 cells and mCherry-expressing *NAT10* KO U251 cells were co-cultured in the same soft agar plate. Even under the same external conditions, spheroid formation of *NAT10* KO U251 cells (Fig. S1) suggests that NAT10 contributes to the cancer stemness of GBM without being influenced by extracellular stimuli.

To identify the intracellular biological pathways whose gene expressions are under the control of NAT10, we focused on NAT10-regulated 393 genes (Table S2) and conducted the ChIP-Atlas enrichment analysis (28, 29) (https://chip-atlas.org). A variety of transcriptional factors were identified as being under the control of NAT10 (Table S3). In addition, we also performed wiki_pathway analysis using these enriched transcriptional factors (30). The results of pathway analysis revealed regulation of sister chromatid separation at the metaphase-anaphase transition (WP4240) and interaction of polycomb repressive complex 2 (WP2916) were significantly enriched in *NAT10* KO U251 cells (Fig. 3*B*). By integrating the current pathway analysis with NAT10 target genes from previous RNA-seq datasets (GSE162043 for RedaC:T-seq and GSE102113 for acRIP-seq), we identified several candidate genes potentially undergoing acetylation by NAT10, including MAD1L1, a regulator of sister chromatid segregation, and JARID2, SUZ12, and THRAP3, which are associated with PRC2 binding factors (Fig. 3*C*). Among them, JARID2 protein levels decreased in *NAT10* KO U251 cells (Fig. 3*D*). Consequently, we further focused on this PRC2 component as a key NAT10 target and investigated its function in regulating GBM stemness. To determine whether JARID2 is involved in the regulation of GBM stemness, we prepared JARID2-expressing *NAT10* KO U251 cells and conducted spheroid formation assay. The spheroid formation ability of *NAT10* KO U251 cells was significantly restored by enhanced expression of JARID2 (Fig. 3*E*), suggesting that NAT10 contributes to the maintenance of GBM malignancy through regulating JARID2 expression.

### NAT10 stabilizes JARID2 mRNA through ac4C modification

**Figure 4:**
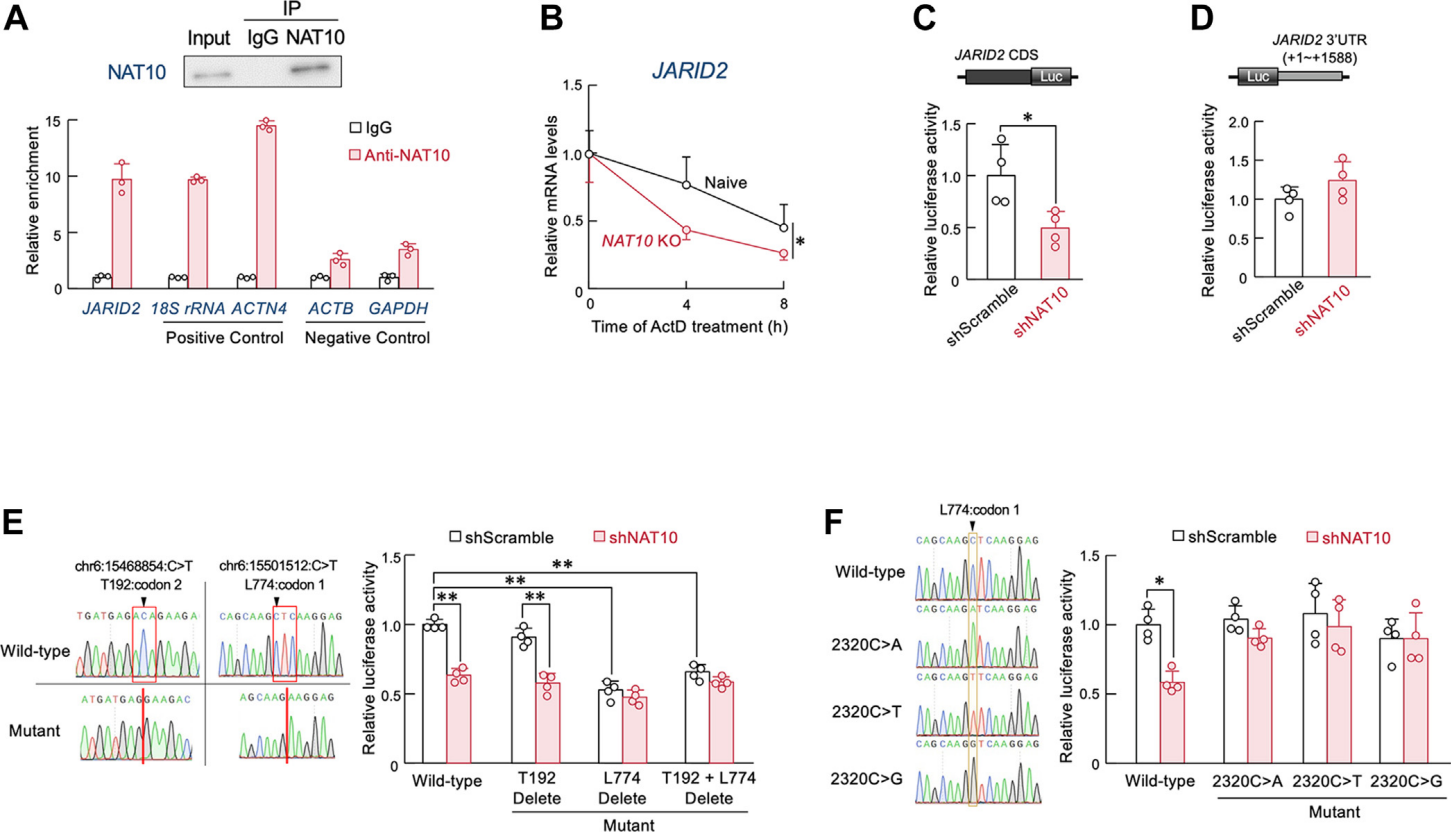
**NAT10 regulates *JARID2* mRNA stability by acetylation of 2320 cytidine residue in *JARID2* CDS**. *A*, RNA precipitation (RIP) assay using anti-NAT10 antibodies. *Upper* panel shows western blotting analysis of NAT10 in immune-precipitates by anti-NAT10 antibodies. *Lower* graph shows quantitative real-time RT-PCR analysis of NAT10-RIP using primers listed in Table 1. Values are the mean with S.D. (n = 3). *B*, difference in the stability of *JARID2* mRNA between naive and *NAT10* knockout (KO) U251 cells. The mRNA levels were normalized by *ATP5E* mRNA levels. Basal levels of expression (0 h; the time of the initiation of ActD treatment) was set at 1.0. Values show the mean with S.D. (n = 4). ∗; *p* < 0.05 significant difference between the two groups. (*F*_1,17_ = 5.936, *p* = 0.026; Two-way ANOVA with the Tukey-Kramer *post hoc* test). *C*, The reporter activity of *JARID2* CDS::Luc in U251 cells transfected with shRNA against *NAT10*. Control cells were transfected with scramble shRNA (shScramble). Schematic diagram of *JARID2* CDS::Luc is shown in the *top* of panels. Values of firefly luciferase activity were normalized to renilla luciferase activity. Values show the mean with S.D. (n = 4). ∗; *p* < 0.05 significant difference between the two groups (*t*_6_ = 3.006, Student’s *t* test). *D*, the reporter activity of *JARID2* 3′-UTR::Luc in U251 cells transfected with shRNA against *NAT10*. Schematic diagram of *JARID2* 3′-UTR::Luc is shown in the *top* of panel. Control cells were transfected with shScramble. Values of firefly luciferase activity were normalized to renilla luciferase activity. Values show the mean with S.D. (n = 4). (*t*_6_ = 1.670, Student’s *t* test). *E*, *left* panel shows Sanger sequencing of wild-type and mutated (T192 and L774 deletion) *JARID2* CDS::Luc. *Black* arrowheads indicate the NAT10-mediated acetylation site identified from RedaC:T-seq (GSE162043). *Right* graph shows the reporter activity of wild-type *JARID2* CDS::Luc, *JARID2* T192 del::Luc, *JARID2* L774 Del::Luc, and *JARID2* T192 + L774 del::Luc in U251 cells transfected with shRNA against *NAT10*. Control cells were transfected with scramble shRNA. Luciferase activities of shScramble-transfected cells are set at 1.0. Values of firefly luciferase activity were normalized to renilla luciferase activity and show the mean with S.D. (n = 4). ∗∗; *p* < 0.01 significant difference between the two groups. (*F*_7,24_ = 46.719, *p* < 0.001, ANOVA with the Tukey-Kramer *post hoc* test). *F,Left* panel shows Sanger sequencing of wild-type and point mutated (2320C > A, T, or G) *JARID2* CDS::Luc. *Right* graph shows the reporter activity of wild-type *JARID2* CDS::Luc, *JARID2* 2320C > A::Luc, *JARID2* 2320C > T::Luc, and *JARID2* 2320 C > G::Luc in U251 cells transfected with shRNA against *NAT10*. Control cells were transfected with scramble shRNA. Luciferase activities of shScramble-transfected cells are set at 1.0. Values of firefly luciferase activity were normalized to renilla luciferase activity and show the mean with S.D. (n = 4). ∗; *p* < 0.05 significant difference between the two groups. (*F*_7,24_ = 4.357, *p* = 0.003, ANOVA with the Tukey-Kramer *post hoc* test).

NAT10 is an RNA acetyltransferase that binds to mRNA and catalyzes the acetylation of cytidine residues. To investigate whether NAT10 protein binds to *JARID2* mRNA, we performed RNA immunoprecipitation assay using anti-NAT10 antibodies. *JARID2* mRNA was co-precipitated with anti-NAT10 antibodies (Fig. 4*A*). The amount of *JARID2* mRNA precipitated with anti-NAT10 antibodies, relative to that with control IgG, was comparable to the levels observed for 18S rRNA and *ACTN4* mRNA, both of which have been previously reported to undergo acetylation (31). In contrast, the enrichment levels of *ACTB* and *GAPDH* mRNAs—whose cytidine residues are not acetylated by NAT10 (12)—were lower than that of *JARID2* mRNA. These results suggest that NAT10 protein interacts with *JARID2* mRNA, potentially for cytidine acetylation. NAT10 has been reported to promotes mRNA stability by ac4C modification in the coding sequence (CDS) and 3′-untranslated region (UTR) (12). Therefore, we investigated how NAT10 regulates the expression of JARID2 protein by focusing on its mRNA stability. After treatment of naive and *NAT10* KO U251 cells with Actinomycin D (ActD), an inhibitor of RNA synthesis, we assessed *JARID2* mRNA levels at 4 h intervals. The stability of JARID2 mRNA was significantly reduced in *NAT10* KO U251 cells (Fig. 4*B*). The half-lives of *JARID2* mRNA in naive and *NAT10* KO U251 cells were estimated approximately 7.09 h and 4.57 h, respectively.

Next, we explored whether NAT10-mediated stability of *JARID2* mRNA is caused by the acetylation of CDS or 3′-UTR. We constructed two reporter vectors. One vector was designed with a luciferase gene inserted downstream of the human *JARID2* mRNA CDS (*JARID2* CDS::Luc), while other one was designed in which the human *JARID2* mRNA a 3′-UTR was inserted downstream of the luciferase gene (*JARID2* 3′-UTR::Luc). The reporter activity of *JARID2* CDS::Luc was significantly decreased by down-regulation of NAT10 (Fig. 4*C*), but the reporter activity of *JARID2* 3′-UTR::Luc in *NAT10* knockdown cells was comparable to that observed in control cells (Fig. 4*D*). These results suggest that NAT10 stabilizes the *JARID2* mRNA through acetylation of its CDS.

We also searched for NAT10-mediated acetylated cytidine residues (ac4C) on the *JARID2* mRNA CDS using RedaC:T-seq dataset (GSE162043). Two ac4C sites were identified within the *JARID2* mRNA CDS, located at the codons for the 192nd threonine (T192) and the 774th leucine (L774). Therefore, we constructed reporter vectors in which the codons corresponding to the NAT10-mediated acetylated cytidine residues at positions T192 (*JARID2* T192 del::Luc), L774 (*JARID2* L774 del::Luc), or both T192 and L774 (*JARID2* T192 + L774 del::Luc) were deleted from the JARID2 CDS::Luc vector and subsequently assessed their reporter activity. Deletion of the codons for the T192 cytidine residue had negligible effects on the reporter activity, whereas deletion of codons for the L774 or both T192 and L774 cytidine residues significantly decreased reporter activities (Fig. 4*E*). These results suggest that NAT10 regulates the *JARID2* mRNA stability through cytidine acetylation at the L774 codon. This notion was also supported by the fact that downregulation of NAT10 was unable to further decrease the luciferase activities of *JARID2* L774 del::Luc and *JARID2* T192 + L774 del::Luc. Furthermore, we introduced point mutations at the cytidine residue (position 2320) within the *JARID2* CDS to adenosine, thymidine (uridine), or guanosine, and conducted a luciferase reporter assay using *JARID2* CDS-inserted constructs. The reporter activity of the wild-type *JARID2* CDS::Luc construct was significantly decreased upon NAT10 knockdown. In contrast, reporter activity of the point mutants—2320C > A, 2320C > T, and 2320C > G—showed little to no change following *NAT10* knockdown (Fig. 4*F*). These findings suggest that NAT10 stabilizes *JARID2* mRNA through ac4C modification on the 2320 cytidine residue within *JARID2* CDS.

### JARID2 is involved in the regulation of GBM malignancy

**Figure 5:**
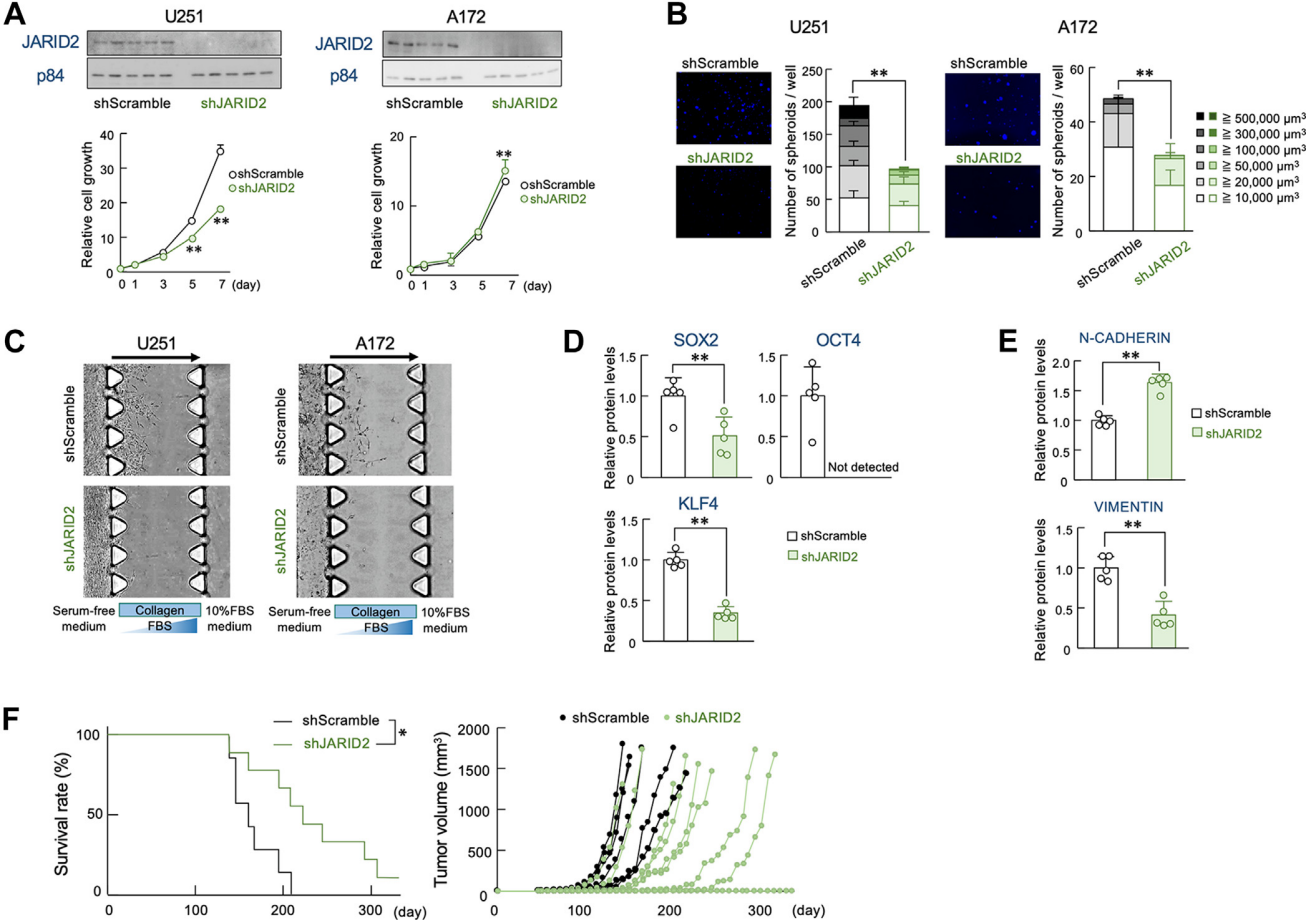
**Contribution of JARID2 to the maintenance of stemness properties of GBM cells**. *A*, decrease in the growth ability of U251 and A172 cells by downregulation of JARID2. Each *upper* panel show JARID2 protein levels in U251 and A172 cells transfected with shRNA against *JARID2* or scramble shRNA (shScramble). Each below graph shows the cell viability of seeding day (day 0) was set at 1.0. Values show the mean with S.D. (n = 6). ∗∗; *p* < 0.01 significant difference from shScramble group at corresponding time points. (*F*_9,50_ = 1636.125 *p* < 0.001 for U251 cells, *F*_9,50_ = 140.977 *p* < 0.001 for A172 cells, ANOVA with the Tukey-Kramer *post hoc* test). *B*, the spheroid formation ability of U251 and A172 cells transfected with shRNA against *JARID2* or shScramble. Eash *left* panel shows a representative photograph of Hoechst33342-stained spheroids formed by shScramble- or shJARID2-transfected U251 and A172 cells. *Right* panel shows the number of spheroids and the distribution of their diameters. Values show the mean with S.D. (n = 5–6 for U251 cells, n = 8 for A172 cells). ∗∗; *p* < 0.01 significant difference between the two groups (*t* = 11.897. for U251 cells, *t* = 3.412 for A172 cells, Welch’s *t* test). *C*, the invasion ability of U251 and A172 cells transfected with shRNA against *JARID2* or shScramble. Microphotographs show invasion of cells into 3D collagen gel. *D*, the protein expression levels of SOX2, OCT4, and KLF4 in U251 cells transfected with shRNA against *JARID2* or scramble shRNA (shScramble). Values of protein levels were normalized to p84 protein levels. Values show the mean with S.D. (n = 5). ∗∗; *p* < 0.01 significant difference between the two groups (*t*_8_ = 3.407 for SOX2, *t*_8_ = 12.072 for KLF4, Student’s *t* test). *E*, the protein expression levels N-CADHERIN and VIMENTIN in shScramble- or shJARID2-transfected U251 cells. Values of protein levels were normalized to those of β-ACTIN levels. Values show the mean with S.D. (n = 5). ∗∗; *p* < 0.01 significant difference between the two groups (*t*_8_ = 9.389 for N-CADHERIN, *t*_8_ = 6.029 for VIMENTIN, Student’s *t* test). *F*, decrease in the malignancy of U251 cells by downregulation of JARID2. shScramble or shJARID2 RNA expressing lentivirus transfected U251 cells were subcutaneously implanted in Balb/c-nude mice. *Left* graph shows Kaplan-Meier survival curves shScramble or shJARID2 RNA expressing lentivirus transfected U251 tumor-bearing mice (n = 7 for shScramble, n = 9 for shJARID2). *Right* graph shows the tumor volume of each individual mouse. ∗; *p* < 0.05 significant difference between the two groups (LogRank Holm-Sidak test).

In the final set of experiments, we investigated whether JARID2 is involved in the regulation of GBM malignancy. Downregulation of JARID2 decreased both the growth and spheroid formation abilities of U251 cells (Fig. 5*A* left and 5B left). While *JARID2* knockdown (KD) had minimal effect on the growth of A172 cells (Fig. 5*A* right), spheroid formation ability was significantly decreased in *JARID2* KD cells (Fig. 5*B* right). The invasive potential of *JARID2* KD U251 cells was attenuated compared to that of control U251 cells (Fig. 5*C* left). A similar decreased invasive potential was also observed in *JARID2* KD A172 cells (Fig. 5*C* right). These data suggest that JARID2 promotes the aggressive properties of GBM. Consistent with the results, the protein levels of SOX2, OCT4, and KLF4 were decreased in *JARID2* KD U251 cells (Fig. 5*D*). Although the protein levels of N-CADHERIN were increased in *JARID2* KD U251 cells, VIMENTIN protein levels were decreased by JARID2 downregulation (Fig. 5*E*). These results suggest the contribution of JARID2 to the malignancy of GBM, which led us to conduct *in vivo* experiment with xenograft mouse model. Survival period of *JARID2* KD U251 cells-bearing mice significantly prolonged compared to that with control U251 cells-bearing mice. Tumor formation and growth were suppressed in *JARID2* KD U251-bearing mice (Fig. 5*F*). Taken together, these data indicate that GBM malignancy is promoted through the upregulation of JARID2.

## Discussion

**Figure 6:**
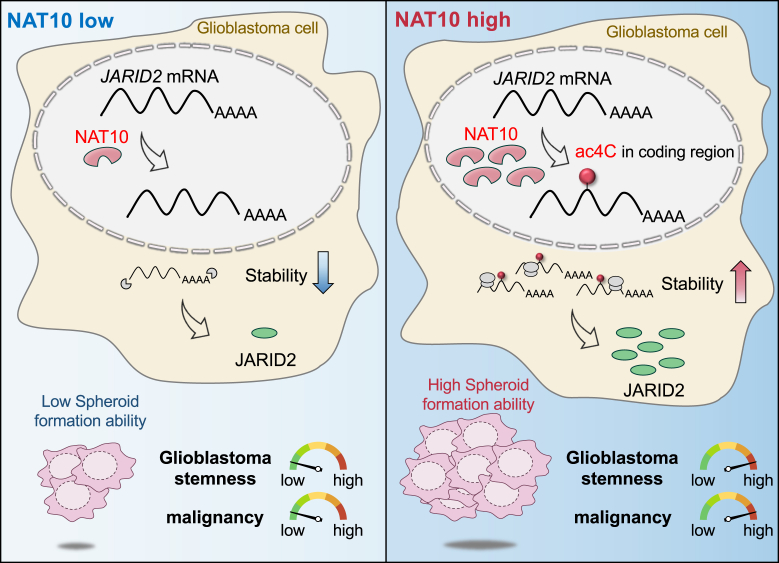
**Schematic diagram of the maintenance of GBM malignancy by NAT10-mediated acetylation of *JARID2* mRNA**. Upregulation of NAT10 leads to acetylation and subsequent stabilization of *JARID2* mRNA. This increased stability enhances JARID2 protein levels, which in turn contributes to the malignant properties of GBM.

NAT10, a multifunctional enzyme, has been recognized as a crucial factor in the complex landscape of cancer biology, with its diverse role in tumorigenesis affecting processes such as cell proliferation, differentiation, survival, and genomic stability maintenance (12). In this study, we elucidated the role of NAT10 in enhancing glioblastoma (GBM) stemness by promoting JARID2 expression. Mechanistically, NAT10 acetylates cytidine residues within the coding sequence of *JARID2* mRNA, leading to increased mRNA stability and enhanced translational efficiency. This results in the upregulation of JARID2, an epigenetic regulator known to promote stemness properties. Collectively, our findings suggest that NAT10 acts as a key regulator of GBM stemness *via* RNA epitranscriptomic modification of *JARID2* mRNA (Fig. 6).

Nuclear factor kappa B (NF-κB) is implicated as a positive regulator to induce the expression of NAT10 in bladder cancer (15). Specifically, p65, a component of NF-κB, binds to the *NAT10* promoter region and enhances its transcriptional activity. Indeed, increased expression of *RELA* mRNA, encoding p65 protein, and the activation of TNF-α-mediated NF-κB signaling are observed in GBM-formed tumor masses (32). We also found a positive correlation between the *NAT10* and *RELA* mRNA expression levels in GBM patients, as revealed by analysis of TCGA_GBM and Rembrandt database (Fig. S2). These facts suggest that the activation of NF-κB signaling, probably due to inflammatory stimuli, contributes to the up-regulation of NAT10 expression in GBM cells.

Ac4C modification is widely observed in the mRNA of human cancer cells. Previous studies have demonstrated that cytidine acetylation in mRNA tends to occur at the third base of the codon (Wobble base). This modification has been shown to enhance mRNA stability and translation efficiency by thermally stabilizing mRNA and tRNA binding (33). Acetylation at the wobble base forms stable wobble base pairs with guanine residues of tRNA anticodons in the ribosome, thereby increasing translation efficiency through stabilization of ribosome binding (12). On the other hand, the NAT10-mediated acetylation site within *JARID2* mRNA was identified at the first residue of the 774th leucine rcodon. Although the molecular significance of cytidine acetylation at the first codon remains unclear, reporter assays using a construct in which the coding sequence of *JARID2* mRNA was fused upstream of the luciferase gene suggest that acetylation at this site promotes both mRNA stability and JARID2 protein expression. Acetylation of 5′-UTR and 3′-UTR has been demonstrated to decrease translational efficiency and mRNA stability, respectively (14, 31). Therefore, it is plausible that acetylation of the cytidine residue at the first codon of the 774th leucine in the coding region of *JARID2* mRNA similarly contributes to enhanced translation efficiency and mRNA stabilization. However, further detailed investigations are required to fully elucidate these mechanisms.

NAT10 enhances the self-renewal capacity by increasing the expression of *POU5F1* mRNA through acetylation in human embryonic stem cells (hESCs) (13). In contrast, acetylation of *POU5F1* mRNA is not observed in human cervical carcinoma (HeLa) cells (12), indicating that the NAT10 targets different mRNA between normal and cancer cells. NAT10-mediated RNA acetylation has been identified not only in mRNA but also in ribosomal RNA (rRNA) and transfer RNA (tRNA). The RNA acetylation is not solely mediated by NAT10, but involves interaction with SNORD13, short nucleolar RNA (snoRNA) (34), and THUMP domain-containing protein 1 (THUMPD1) as an adapter (35). The selection of NAT10 target RNAs is also regulated by these adaptors, suggesting that additional, unidentified NAT10-binding proteins may contribute to the specificity of mRNA acetylation. In addition, ac4C modifications within Kozak sequences of 5′-UTR mRNA inhibit canonical start codon recognition and reduce transcript levels (31), while ac4C modification in *Gremlin1* mRNA accelerates its degradation (36). Further studies are necessary to elucidate the mechanisms underlying NAT10-mediated mRNA acetylation specificity.

NAT10 also regulates the differentiation of hESCs through modulation of the chromatin landscape *via* acetylation of acidic nuclear phosphoprotein 32 B (ANP32 B) mRNA. This study demonstrates that down-regulation of NAT10 increases or decreases the methylation state of the H3K27 in hESCs, probably *via* mediating by PRC2 (37). JARID2 negatively and positively regulates the recruit region of PRC2 and its methylation activity (38, 39). Consistent with the landscape changes of H3K27me3 by down-regulation of NAT10, JARID2-defective ESCs also alter methylation levels of H3K27 and disrupt the normal regulation of self-renewal and differentiation (40). Therefore, JARID2 may contribute to GBM stemness through regulating PRC recruitment and its histone methylation activity on H3K27. This notion is also supported by the previous findings that JARID2 promotes the invasion ability of lung cancer cells by recruiting PRC2 to the promoter region of EMT-regulatory genes CDH1 and microRNA-200 family (41).

On the other hand, JARID2 also causes deacetylation and methylation of target gene promoters and downregulates several tumor suppressor genes, leading to the promotion of proliferation, invasion, and cancer stemness in breast cancer (42). We observed partial suppression of GBM stemness in U251 cells after treatment with EZH1/2 inhibitor valemetostat (Fig. S3). The suppressive effect of valemetostat on spheroid formation of U251 cells was modest compared to that observed in JARID2-downregulated U251 cells (Fig. 5*B* left), suggesting, in addition to PRC2-mediated regulation, other mechanisms may also contribute to the regulation of GBM stemness by JARID2. Previous studies have shown that JARID2 enhances the efficiency and kinetics of reprogramming into iPS cells *via* interactions with PRDM14, ESRRB, and SALL4A, independently of PRC2 (43). Indeed, JARID2 lacking the N-terminus, which is required for interaction with EZH2, retains the ability to induce differentiation genes (44). These results suggest that JARID2 promotes GBM stemness not only through PRC2, but also through other mechanisms. Further investigation is required to elucidate the detailed mechanism of JARID2-mediated enhancement of GBM stemness.

Recent accumulating evidence has demonstrated the involvement of the post-transcriptional modification in promoting tumor aggressiveness (45). Our present findings extend to understanding the role of mRNA acetylation by NAT10 in the maintenance of GBM stemness. The previously unrecognized function of the NAT10-JARID2 cascade in GBM stem cells may provide a novel therapeutic target for the treatment of GBM.

## Materials and methods

### Cell and treatment

U251 and A172 human glioblastoma cells were purchased from the National Institute of Biomedical Innovation (Osaka, Japan). Lenti-X 293T cells were purchased from Takara Bio Inc . U251 and Lenti-X 293T cells were cultured in DMEM (Gibco BRL), and A172 cells were cultured in high-glucose DMEM (FUJIFILM Wako) supplemented with 10% FBS (Bioweat) and 0.5% penicillin-streptomycin solution (FUJIFILM Wako). Cells were maintained at 37^o^C in a humidified 5% CO_2_ atmosphere. Cells were confirmed that there was no microbial contamination using MycoBlue *Mycoplasma* Detector (Vazyme Biotech Co., Ltd). Cells were authenticated by each cell bank using short tandem repeat PCR analysis and were used in less than 3 months from frozen stocks.

### Construction of *NAT10* knockout U251 and A172 cells

sgRNAs targeting exon 5 of the human NAT10 gene for CRISPR knockout were constructed using the Guide-it single-guide RNA (sgRNA) *in Vitro* Transcription Kit (Takara). Cells were transfected with the sgRNA and Guide-it Recombinant Cas9 (Electroporation-ready) (Takara) using the NEPA21 electroporator (Nepagene). Cell clones were isolated and expanded in 96-well plates *via* ultrafiltration.

### Construction of mEGFP-, and mCherry2-expressing U251 cells

mEGFP-N1 plasmids (RRID: addgene_54767) and mCherry2-N1 plasmids (RRID: addgene_54517) were obtained from Addgene. The sequences of mEGFP and mCherry2 were subcloned into pLVSIN-CMV Puro (Takara). Lentiviral particles were produced using the Lentiviral High Titer Packing Mix with pLVSIN series (Clonetech) in Lenti-X 293T-cell lines. mEGFP-expressing and mCherry2-expressing lentivirus particles, along with 10 μg/ml of polybrene (Sigma Aldrich), were added to the U251 cell culture medium and incubated for 24 h. Cells transduced with mEGFP-expressing and mCherry2-expressing lentivirus were selected with 5 μg/ml of puromycin.

### Construction of *JARID2* knockdown U251 and A172 cells

Scramble shRNA (Scramble[shRNA#1]) and JARID2 shRNA (pLV[shRNA]-Puro-U6>hJARID2[shRNA#1]) expressing plasmid were provided by VectorBuilder. Lentiviral particles were generated using the Lentiviral High Titer Packing Mix with pLVSIN series using Lenti-X 293T cell lines. Scramble shRNA and JARID2 shRNA-expressing lentivirus particles, along with 10 μg/ml polybrene, were added to the culture media of U251 and A172 cells and incubated for 24 h. Cells transduced with shScramble and shJARID2 were selected with 5 μg/ml of puromycin.

### Determination of the growth ability of cells

Cells were seeded at a density of 2000 cells per well in 100 μl of culture medium in 96-well plates. Cell viability was assessed on Days 1, 3, 5, and 7 post-seeding using the Cell Titer-Glo luminescent cell viability assay kit (Promega). The growth rate was calculated by dividing the change in cell viability from the basal level (Day 0).

### Spheroid formation assay

The ability of cells to grow in an anchorage-independent manner was assessed to evaluate the spheroid formation. Cells were seeded in soft agar containing DMEM with 10% FBS at a density of 6.0 × 10^3^ cells per well in a 24-well plate. For co-culture experiments, mEGFP-expressing naive U251 cells and mCherry2-expressing *NAT10* knockout U251 cells were seeded in soft agar with DMEM containing 10% FBS at a density of 3.0 × 10^3^ cells each per well in a 24-well plate. On day 10 post-seeding, spheroid formation was evaluated by staining with Hoechst33342 (Dojindo Laboratories). Cells were observed using the KEYENCE all-in-one microscope BZ-X800, and the numbers and size of spheroids were measured by the BZ Analyzer software (KEYENCE).

### Invasion assay

Cells were seeded onto a collagen-filled 3D Cell Culture Chip (AIM BIOTECH, Singapore) according to the manufacturer’s instructions. The culture medium, containing 10% FBS, was replaced every 2 days. On day 14 post-seeding, cells were observed using the KEYENCE all-in-one microscope BZ-X800 (KEYENCE).

### Animals and treatments

Male Balb/c-nude mice were purchased from the Jackson Laboratory Japan (Yokohama, Japan). Mice were housed in groups of 6 to 8 per cage in a light-controlled room (ZT, zeitgeber time; ZT0, lights on, ZT12, lights off) at 24 ± 1°C, with 60 ± 10% humidity, and provided with food and water *ad libitum*. U251 cells (8.0 × 10^5^ cells) suspended in 10 μl of PBS were implanted subcutaneously into the backs of 6-week-old Balb/c-nude mice under isoflurane anesthesia (Pfizer, New York, NY). U251 cells (1.6 × 10^6^ cells), suspended in 5 μl of PBS, were implanted slowly over a 2 min into the brain (a small hole was drilled in the skull at stereotaxic coordinates: 1.0 mm posterior to the bregma, +2.0 mm mediolateral from the midline and 3.0 mm depth) of 6-week-old Balbc-nude mice under isoflurane anesthesia and fixed on a stereotactic frame using SR-5M-HT (NARISHIGE, Tokyo, Japan). All protocols using mice were reviewed and approved by the Animal Care and Use Committee of Kyushu University. All methods were performed in accordance with the relevant guidelines and regulations.

### Quantitative real-time RT-PCR analysis

Total RNA was extracted from cells using RNAiso Plus (Takara Bio Inc.) according to the manufacturer’s instructions. For quantitative real-time RT-PCR, the cDNA equivalent of 10 ng of RNA was amplified by PCR using the LightCycler 96 system (Roche Diagnostics) with THUNDERBIRD SYBR qPCR Mix (TOYOBO). Sequences of primers are listed in Table 1. We confirmed no significant amplification of RNA products without reverse transcription. The PCR-amplified products were separated by electrophoresis using 1% agarose gel containing ethidium bromide. Signals from the agarose gel were detected using LAS3000 (FUJIFILM).

**Table 1:** Primer sets for quantitative RT-PCR analysis

Gene	Primer sequence
Human *JARID2*	
Forward	5′-ACCAGTCTAAGGGATTAGGACC-3′
Reverse	5′-TGCTGGGACTATTCGGCTGA-3′
Human *18S rRNA*	
Forward	5′-CGGCTACCACATCCAAGGAA-3′
Reverse	5′-GCTGGAATTACCGCGGCT-3′
Human *ACTN4*	
Forward	5′-GCAGCATGGGCGACTACAT-3′
Reverse	5′-TTGAGCCCGTCTCGGAAGT-3′
Human *ACTB*	
Forward	5′-AAACTGGAACGGTGAAGGTG-3′
Reverse	5′-CGCATCTCATATTTGGAATGACT-3′
Human *GAPDH*	
Forward	5′-ACAACTTTGGTATCGTGGAAGG-3′
Reverse	5′-GCCATCACGCCACAGTTTC-3′
Human *ATP5E*	
Forward	5′-GTGGCCTACTGGAGACAGG-3′
Reverse	5′-GGAGTATCGGATGTAGCTGAGT-3′
Human *JARID2* [1.0 kbp]	
Forward	5′-GCTGAACGGACACGTGAAGAA-3′
Reverse	5′-TGGTGGTCGTTCTCTGTGTGG-3′

### Western blotting

Nuclear and cytosolic fractions from U251 cells were prepared by centrifugation. Total protein was extracted using lysis buffer [20 mmol/L Tris-HCl (pH 7.4), 150 mmol/L NaCl, 0.1% SDS, 1% Nonidet P-40, 0.5% Deoxycholic acid, and 2 mmol/L EDTA]. The extracts were centrifuged at 15,000×*g* for 10 min at 4 ^o^C, and the supernatant was collected. Protein extracts were mixed with 2 × sample buffer [250 mmol/L Tris-HCl (pH 6.8), 2% (w/v) SDS, 30% (v/v) glycerol, 10% (v/v) 2-mercaptoethanol, 0.01% (w/v) Bromophenol Blue] and denatured at 95 ^o^C for 5 min. Nuclear protein was extracted using cell lysis buffer [150 mmol/L NaCl, 50 mM HEPES-NaOH (pH 7.4), 1% (v/v) NP-40, 1 mol/L Hexylene glycol]. The extracts were centrifuged at 500×*g* for 10 min at 4 ^o^C, and the pellet was washed once with cell lysis buffer. After washing, the pellet was extracted using nuclear lysis buffer [150 mmol/L NaCl, 50 mmol/L HEPES-NaOH (pH 7.4), 0.5% (w/v) sodium deoxycholate, 0.5%(w/v) SDS, 1 mol/L Hexylene glycol].

Protein extracts were mixed with 2 × sample buffer and denatured at 95 ^o^C for 5 min. The samples were separated by SDS-PAGE and transferred to a polyvinylidene difluoride membrane. Membranes were reacted with antibodies against NAT10 (13365-1-AP, Proteintech, Wuhan, China, RRID:AB_2148944), p84 (THOC1, 10920-1-AP, Proteintech, RRID:AB_2202239), SOX2 (AF2018, R&D systems, RRID:AB_355110), KLF4 (#4038, Cell Signaling Technology, RRID:AB_2265207), OCT4 (#4038, Cell Signaling Technology), N-CADHERIN (AF6426, R&D systems, RRID:AB_10718850), VIMENTIN (MAB21052, R&D systems, RRID:AB_2832972), SUZ12 (#3737, Cell Signaling Technology, RRID:AB_2196850), JARID2 (#13594, Cell Signaling Technology, RRID:AB_2798269), THRAP3 (TRAP150, sc-133250, Santa Cruz Biotechnology, RRID:AB_2202901), MAD1L1 (MAD1, 18322-1-AP, Proteintech, RRID:AB_2139251) and β-ACTIN conjugated with horseradish peroxidase (sc-47778, Santa Cruz Biotechnology, RRID:AB_2202239).

Specific antigen–antibody complexes were visualized using HRP-conjugated anti-rabbit antibody (ab97051, Abcam, RRID:AB_10679369), HRP-conjugated anti-goat antibody (sc-2020, Santa Cruz Biotechnology, RRID:AB_631728), HRP-conjugated anti-mouse antibody (ab6820, Abcam, RRID:AB_955438), HRP-conjugated anti-sheep antibody (sc-2770, Santa Cruz Biotechnology, RRID:AB_656968) and ImmunoStar LD (FUJIFILM Wako). Visualized images were scanned using ImageQuant LAS4000 (GE Healthcare). The band intensity of Western blotting was quantified using Image J (version 1.8.0, NIH) with the “Gel Analysis” function to measure peak areas (46). The obtained data were normalized to the housekeeping protein, β-ACTIN and p84.

### Luciferase reporter assay

Reporter vectors were constructed using the pGL4.13 reporter plasmids. The coding region of the human *JARID2* mRNA was inserted N-terminal of the luciferase gene (*JARID2* CDS::Luc). The 3′-untranslated region (3′-UTR) of the human *JARID2* mRNA was inserted downstream of the luciferase gene (*JARID2* 3′-UTR::Luc). Deletions were introduced into the CDS of the human *JARID2* gene at the codon for the 192nd threonine (*JARID2* T192 del::Luc) and the 774th leucine (*JARID2* L774 del::Luc) as well as at both codons simultaneously (*JARID2* T192 + L774 del::Luc). Moreover, point mutations were introduced into the cytidine located on 2320 in its CDS for adenosine (*JARID2* 2320C > A::Luc), for thymidine (Uridine) (*JARID2* 2320C > T::Luc), and for guanosine (*JARID2* 2320C > G::Luc).

U251 cells were seeded on 24 well culture plates at 1.0 × 10^5^ per well. Cells were transfected with 300 ng of each reporter construct: *JARID2* CDS::Luc, *JARID2* T192 del::Luc, *JARID2* L774 del::Luc, *JARID2* T192 + L774 del::Luc, *JARID2* 2320C > A::Luc, *JARID2* 2320C > T::Luc, *JARID2* 2320C > G::Luc, and *JARID2* 3′UTR::Luc. A total of 5 ng of phRL-TK vector (Promega) was also transfected as an internal control reporter. Cells were harvested for 24 h post-transfection, and lysates were analyzed using the Dual-Luciferase reporter assay system (Promega). The ratio of fireflies to renilla luciferase activity in each sample served as a measure of normalized luciferase activity.

### RNA immunoprecipitation (RIP)

Cells were lysed with 1 ml of nuclear isolation buffer [1.28 M sucrose, 40 mM Tris-HCl (pH 7.5), 20 mM MgCl_2_, and 4% Triton X-100]. After the addition of 1 ml of PBS and 3 ml of RNase-free water to lysates, they were centrifuged at 2500×*g* for 15 min at 4 ^o^C and resuspended nuclear pellet in RIP buffer [150 mM KCl, 25 mM Tris-HCl (pH 7.4), 5 mM EDTA, 0.5 mM dithiothreitol, 0.5% Nonidet P-40]. The resuspended nuclei were homogenized and centrifuged at 15,000×*g* for 10 min, and the supernatant was split into two fractions and was then incubated with anti-NAT10 antibody (13365-1-AP, Proteintech) or normal rabbit IgG (PM035, MBL, Tokyo, Japan) for 2 h at 4 ^o^C with gentle rotation, followed by incubation with protein G magnetic beads (Dynabeads Protein G for IP; Fisher Scientific) for 1 h at 4 ^o^C. Then, the samples were applied magnetic field to pull beads to the side of the tube to remove supernatant and were washed of RIP buffer twice and of PBS once. The beads were used for protein elution while the rest was subjected to RNA extraction using RNAiso Plus (Takara Bio Inc.) and quantitative real-time RT-PCR analysis was performed as mentioned above.

### RNA-seq analysis

Total RNA was extracted from naive and *NAT10* KO U251 cells using the QIAGEN RNeasy Mini Kit (QIAGEN, Hilden, German). The quality of the extracted total RNA was assessed using Novogene’s quality control (QC) report. mRNA was purified from total RNA using poly-T oligo-attached magnetic beads. After fragmentation, the first-strand cDNA was synthesized using random hexamer primers followed by the second strand cDNA synthesis. The library was prepared through end repair, A-tailing, adapter ligation, size selection, amplification, and purification. The quality and quantity of the library were assessed using with Qubit and real-time PCR, while size distribution detection was evaluated with a bioanalyzer. For RNA-sequencing (RNA-seq) analysis, mRNA sequencing was conducted on the Novaseq 6000 platform (PE150, 6 Gb). Raw reads were processed to remove adaptor contaminants and low-quality bases. The cleaned reads were aligned to Human Genome Reference (GRCh38) using STAR, and uniquely mapped reads were quantified using RSEM with default parameters. Gene expression normalization was calculated as Trancripts per kilobase million (TPM).

### Enrichment analysis using ChIP-Atlas

Transcriptional regulatory factors were extracted by enrichment analysis using ChIP-Atlas. We performed on the promoter region (−5000 bp ∼ +100 bp; the distance from the transcription site (+1)) of each gene. Transcriptional regulatory factors with high binding on the altered gene promoters were carried out, as extraction conditions set; Fold enrichment ≧ 1.5 and log Q-value ≦ −1.5.

### Statistical and data analyses

All statistical analyses were conducted using JMP pro 17 software (SAS Institute). Prior to performing ANOVA, data were assessed for normality and homogeneity of variances. The comparison of multiple groups was evaluated using one-way ANOVA followed by Tukey-Kramer *post hoc* test, or Kruskal-Wallis test with Mann-Whitney *U* test. The comparison of two groups was analyzed using either Student’s *t* test, Welch’s *t* test, or Mann–Whitney *U* test. The comparison of Kaplan-meier survival curves data was assessed by LogRank Holm-Sidak test. Correlations between continuous variables were evaluated by Pearson correlation analyses. It was considered to be significant if *p* value was <0.05.

## Data availability

All data supporting the results of the present study are included in the article.

## Supporting information

This article contains supporting information.

## Conflicts of interest

The authors declare that they have no conflicts of interest with the contents of this article.

## References

[1] Angom R.S., Nakka N.M.R., Bhattacharya S. (2023). Advances in glioblastoma therapy: an update on current approaches. Brain. Sci..

[2] Miller K.D., Ostrom Q.T., Kruchko C., Patil N., Tihan T., Cioffi G. (2021). Brain and other central nervous system tumor statistics, 2021. CA A Cancer J. Clini..

[3] Chen J., Li Y., Yu T.-S., McKay R.M., Burns D.K., Kernie S.G. (2012). A restricted cell population propagates glioblastoma growth after chemotherapy. Nature.

[4] Wang X., Prager B.C., Wu Q., Kim L.J.Y., Gimple R.C., Shi Y. (2018). Reciprocal signaling between glioblastoma stem cells and differentiated tumor cells promotes malignant progression. Cell. Stem. Cell..

[5] Atkins I., Kinnersley B., Ostrom Q.T., Labreche K., Il’yasova D., Armstrong G.N. (2019). Transcriptome-wide association study identifies New candidate susceptibility genes for glioma. Cancer. Res..

[6] Patel A.P., Tirosh I., Trombetta J.J., Shalek A.K., Gillespie S.M., Wakimoto H. (2014). Single-cell RNA-seq highlights intratumoral heterogeneity in primary glioblastoma. Science.

[7] Boo S.H., Kim Y.K. (2020). The emerging role of RNA modifications in the regulation of mRNA stability. Exp. Mol. Med..

[8] Roundtree I.A., Evans M.E., Pan T., He C. (2017). Dynamic RNA modifications in gene expression regulation. Cell.

[9] Begik O., Lucas M.C., Liu H., Ramirez J.M., Mattick J.S., Novoa E.M. (2020). Integrative analyses of the RNA modification machinery reveal tissue- and cancer-specific signatures. Genome. Biol..

[10] Li P., Wang W., Zhou R., Ding Y., Li X. (2023). The m5C methyltransferase NSUN2 promotes codon-dependent oncogenic translation by stabilising tRNA in anaplastic thyroid cancer. Clin. Translational. Med..

[11] Su J., Wu G., Ye Y., Zhang J., Zeng L., Huang X. (2021). NSUN2-mediated RNA 5-methylcytosine promotes esophageal squamous cell carcinoma progression via LIN28B-dependent GRB2 mRNA stabilization. Oncogene.

[12] Arango D., Sturgill D., Alhusaini N., Dillman A.A., Sweet T.J., Hanson G. (2018). Acetylation of cytidine in mRNA promotes translation efficiency. Cell.

[13] Liu R., Wubulikasimu Z., Cai R., Meng F., Cui Q., Zhou Y. (2023). NAT10-mediated N4-acetylcytidine mRNA modification regulates self-renewal in human embryonic stem cells. Nucleic. Acids. Res..

[14] Zhang Y., Jing Y., Wang Y., Tang J., Zhu X., Jin W.-L. (2021). NAT10 promotes gastric cancer metastasis via N4-acetylated COL5A1. Sig. Transduct. Target. Ther..

[15] Xie R., Cheng L., Huang M., Huang L., Chen Z., Zhang Q. (2023). NAT10 drives cisplatin chemoresistance by enhancing ac4C-associated DNA repair in bladder cancer. Cancer. Res..

[16] Mughal A.A., Grieg Z., Skjellegrind H., Fayzullin A., Lamkhannat M., Joel M. (2015). Knockdown of NAT12/NAA30 reduces tumorigenic features of glioblastoma-initiating cells. Mol. Cancer..

[17] Mohammad F., Weissmann S., Leblanc B., Pandey D.P., Højfeldt J.W., Comet I. (2017). EZH2 is a potential therapeutic target for H3K27M-mutant pediatric gliomas. Nat. Med..

[18] Holoch D., Margueron R. (2017). Mechanisms regulating PRC2 recruitment and enzymatic activity. Trends. Biochem. Sci..

[19] Peng J.C., Valouev A., Swigut T., Zhang J., Zhao Y., Sidow A. (2009). Jarid2/Jumonji coordinates control of PRC2 enzymatic activity and target gene occupancy in pluripotent cells. Cell.

[20] Kinkel S.A., Galeev R., Flensburg C., Keniry A., Breslin K., Gilan O. (2015). Jarid2 regulates hematopoietic stem cell function by acting with polycomb repressive complex 2. Blood.

[21] Fu Y., Xu J.-J., Sun X.-L., Jiang H., Han D.-X., Liu C. (2017). Function of JARID2 in bovines during early embryonic development. PeerJ.

[22] Bowman R.L., Wang Q., Carro A., Verhaak R.G.W., Squatrito M. (2017). GlioVis data portal for visualization and analysis of brain tumor expression datasets. Neuro Oncol.

[23] Clarke M.F., Dick J.E., Dirks P.B., Eaves C.J., Jamieson C.H.M., Jones D.L. (2006). Cancer stem cells—Perspectives on current Status and Future Directions: AACR Workshop on cancer stem cells. Cancer. Res..

[24] Takahashi K., Yamanaka S. (2006). Induction of pluripotent stem cells from mouse embryonic and Adult Fibroblast cultures by defined factors. Cell.

[25] Lopez-Bertoni H., Johnson A., Rui Y., Lal B., Sall S., Malloy M. (2022). Sox2 induces glioblastoma cell stemness and tumor propagation by repressing TET2 and deregulating 5hmC and 5mC DNA modifications. Sig. Transduct. Target. Ther..

[26] Zhou Y., Zhou B., Pache L., Chang M., Khodabakhshi A.H., Tanaseichuk O. (2019). Metascape provides a biologist-oriented resource for the analysis of systems-level datasets. Nat. Commun..

[27] Matsunaga N., Ogino T., Hara Y., Tanaka T., Koyanagi S., Ohdo S. (2018). Optimized Dosing Schedule based on Circadian Dynamics of mouse breast cancer stem cells Improves the Antitumor effects of Aldehyde Dehydrogenase inhibitor. Cancer. Res..

[28] Oki S., Ohta T., Shioi G., Hatanaka H., Ogasawara O., Okuda Y. (2018). ChIP-Atlas: a data-mining suite powered by full integration of public ChIP-seq data. EMBO Rep..

[29] Zou Z., Ohta T., Oki S. (2024). ChIP-Atlas 3.0: a data-mining suite to explore chromosome architecture together with large-scale regulome data. Nucleic. Acids. Res..

[30] Chen E.Y., Tan C.M., Kou Y., Duan Q., Wang Z., Meirelles G.V. (2013). Enrichr: interactive and collaborative HTML5 gene list enrichment analysis tool. BMC Bioinformatics.

[31] Arango D., Sturgill D., Yang R., Kanai T., Bauer P., Roy J. (2022). Direct epitranscriptomic regulation of mammalian translation initiation through N4-acetylcytidine. Mol. Cell.

[32] Ahsan H., Malik S.I., Shah F.A., El-Serehy H.A., Ullah A., Shah Z.A. (2023). Celecoxib Suppresses NF-κB p65 (RelA) and TNFα expression signaling in glioblastoma. J. Clin. Med..

[33] Hanson G., Coller J. (2018). Codon optimality, bias and usage in translation and mRNA decay. Nat. Rev. Mol. Cell Biol.

[34] Thalalla Gamage S., Bortolin-Cavaillé M.-L., Link C., Bryson K., Sas-Chen A., Schwartz S. (2022). Antisense pairing and SNORD13 structure guide RNA cytidine acetylation. RNA.

[35] Sharma S., Langhendries J.-L., Watzinger P., Kötter P., Entian K.-D., Lafontaine D.L.J. (2015). Yeast Kre33 and human NAT10 are conserved 18S rRNA cytosine acetyltransferases that modify tRNAs assisted by the adaptor Tan1/THUMPD1. Nucleic. Acids. Res..

[36] Zhu Z., Xing X., Huang S., Tu Y. (2021). NAT10 promotes Osteogenic differentiation of mesenchymal stem cells by mediating N4-acetylcytidine modification of Gremlin 1. Stem. Cells. Int..

[37] Hu Z., Lu Y., Cao J., Lin L., Chen X., Zhou Z. (2024). *N* -acetyltransferase NAT10 controls cell fates via connecting mRNA cytidine acetylation to chromatin signaling. Sci. Adv..

[38] Laugesen A., Højfeldt J.W., Helin K. (2016). Role of the polycomb repressive complex 2 (PRC2) in transcriptional regulation and cancer. Cold. Spring. Harb. Perspect. Med..

[39] Pasini D., Cloos P.A.C., Walfridsson J., Olsson L., Bukowski J.-P., Johansen J.V. (2010). JARID2 regulates binding of the Polycomb repressive complex 2 to target genes in ES cells. Nature.

[40] Landeira D., Bagci H., Malinowski A.R., Brown K.E., Soza-Ried J., Feytout A. (2015). Jarid2 coordinates Nanog expression and PCP/Wnt signaling required for efficient ESC differentiation and early Embryo development. Cell. Rep..

[41] Tange S., Oktyabri D., Terashima M., Ishimura A., Suzuki T. (2014). JARID2 is involved in transforming growth factor-Beta-Induced epithelial-mesenchymal transition of lung and Colon cancer cell lines. PLoS One.

[42] Liu W., Zeng Y., Hao X., Wang X., Liu J., Gao T. (2023). JARID2 coordinates with the NuRD complex to facilitate breast tumorigenesis through response to adipocyte-derived leptin. Cancer. Commun..

[43] Iseki H., Nakachi Y., Hishida T., Yamashita-Sugahara Y., Hirasaki M., Ueda A. (2016). Combined overexpression of JARID2, PRDM14, ESRRB, and SALL4A dramatically improves efficiency and kinetics of reprogramming to induced pluripotent stem cells. Stem. Cells..

[44] Al-Raawi D., Jones R., Wijesinghe S., Halsall J., Petric M., Roberts S. (2019). A novel form of JARID2 is required for differentiation in lineage-committed cells. EMBO J..

[45] Barbieri I., Kouzarides T. (2020). Role of RNA modifications in cancer. Nat. Rev. Cancer..

[46] Schneider C.A., Rasband W.S., Eliceiri K.W. (2012). NIH Image to ImageJ: 25 years of image analysis. Nat. Methods..

