# Supplementary material for: N-acetyltransferase 10 promotes glioblastoma malignancy via mRNA stabilization of jumonji and AT-rich interaction domain containing 2
Source: J Biol Chem. 2025 Apr 25;301(6):108544. doi: 10.1016/j.jbc.2025.108544 (PMC12152616; doi:10.1016/j.jbc.2025.108544)
Supplement: Supporting Materials [file mmc1.docx]

**Supplementary Figures**

**N-acetyltransferase 10 promotes glioblastoma malignancy via mRNA stabilization of Jumonji and AT-rich interaction domain containing 2**

Takuto Inoki, Akito Tsuruta, Yoshinori Masakado, Yuichiro Kai, Yuya Yoshida,

Naoya Matsunaga, Shigehiro Ohdo, and Satoru Koyanagi

**Supplementary Figure S1** Spheroid assay under co-culture conditions of naive and *NAT10* knockout U251 cells.

**Supplementary Figure S2** Correlation between NAT10 and RELA expression levels.

**Supplementary Figure S3** Suppression of stemness by pharmacological inhibition of PRC2.

**Supplementary Figure S4** Unedited full blots of Figure 2A

**Supplementary Figure S5** Unedited full blots of Figure 2E

**Supplementary Figure S6** Unedited full blots of Figure 2F

**Supplementary Figure S7** Unedited full blots of Figure 3D

**Supplementary Figure S8** Unedited full blots of Figure 4A

**Supplementary Figure S9** Unedited full blots of Figure 5A

**Supplementary Figure S10** Unedited full blots of Figure 5D

**Supplementary Figure S11** Unedited full blots of Figure 5E


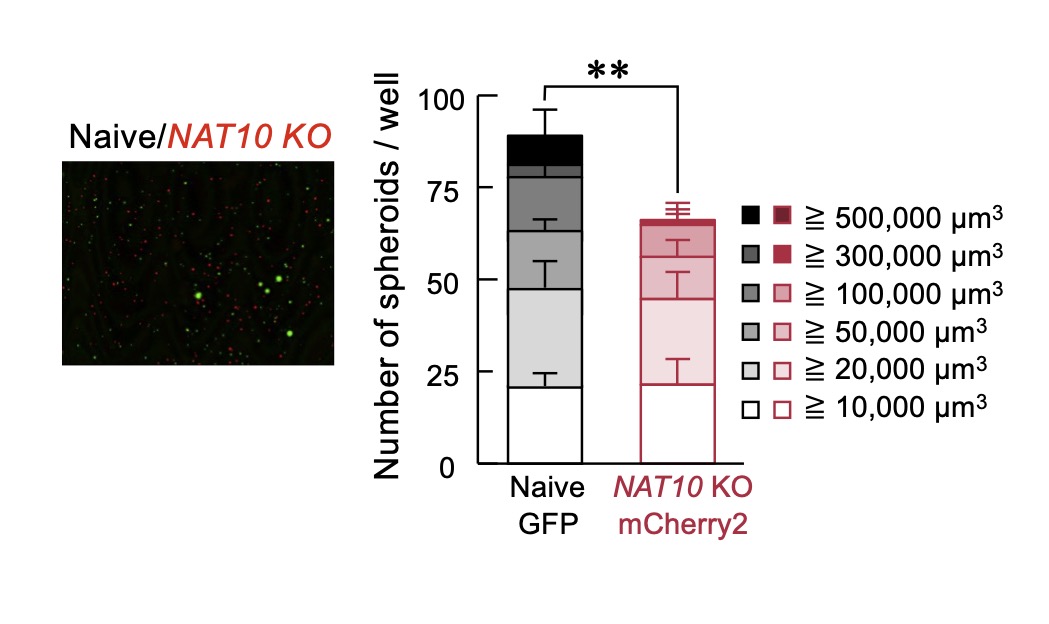


**Supplementary Figure S1 Spheroid assay under co-culture conditions of naive and *NAT10* knockout U251 cells.** The spheroid formation ability under co-culturing with naive and *NAT10* knockout (KO) U251 cells. The left panel shows a representative photograph of the spheroids formed by GFP-expressing naive U251 cells and mCherry2-expressing *NAT10* KO U251 cells. The right panel shows the number of spheroids and the distribution of their diameters. Values show the mean with S.D. (n=3). **; *P*<0.01 significant difference between the two groups (*t*=4.774, Welch’s *t*-test).

**
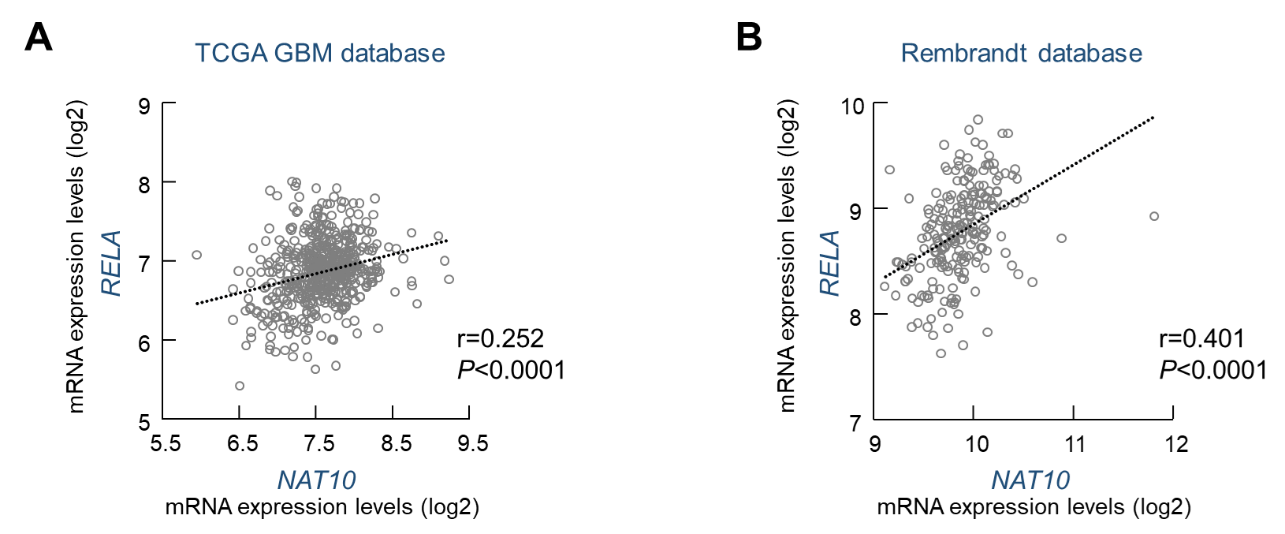
**

**Supplementary Figure S2 Correlation between NAT10 and RELA expression levels.**

**(A and B)** The data from glioblastoma (GBM) patients were obtained from the TCGA GBM database (HG-U133A) (A) and the Rembrandt database (B) and analyzed using GlioVis. Correlation plot shows *NAT10* and *RELA* mRNA expression levels. Pearson correlation coefficients and their *P* values were calculated using JMP pro 17.


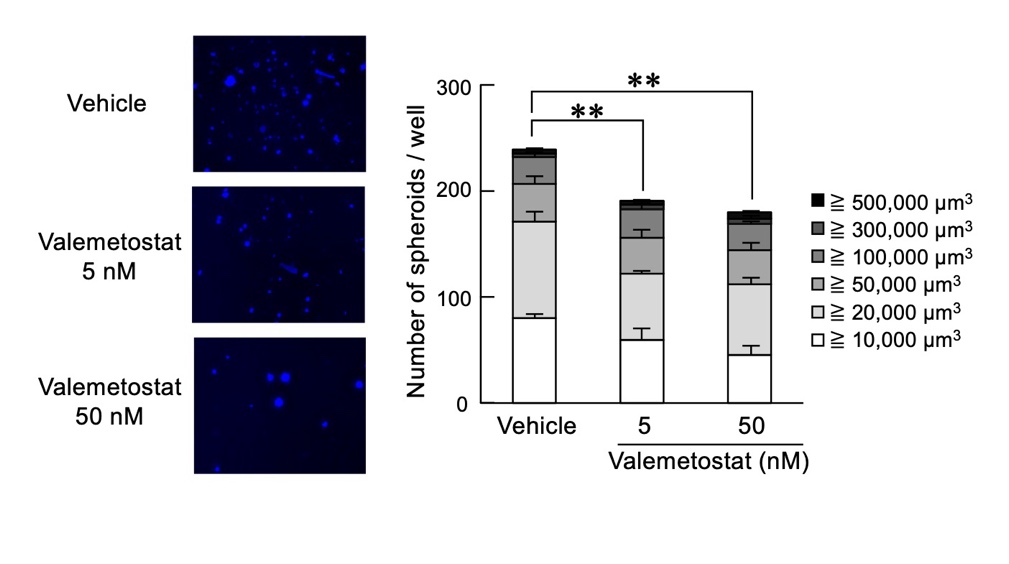


**Supplementary Figure S3 Suppression of stemness by pharmacological inhibition of PRC2.** Spheroid formation ability of U251 cells after treatment with valemetostat, an EZH1/2 inhibitor, at indicating concentrations. Left panel shows a representative photograph of the spheroids formed by U251 cells after treatment with valemetostat. Right panel shows the number of spheroids and the distribution of their diameters. Values show the mean with S.D. (n=4). **; *P*<0.01 significant difference between the two groups (Kruskal-Wallis test with Mann-Whitney U test).

**
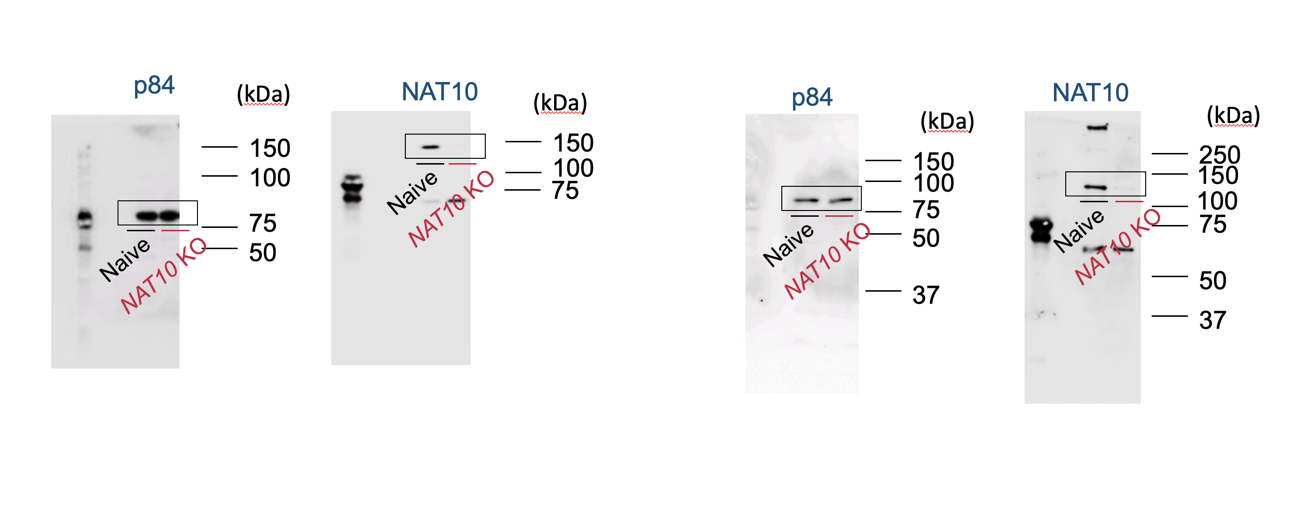
**

**Supplementary Figure S4 Unedited full blots of Figure 2A**

**
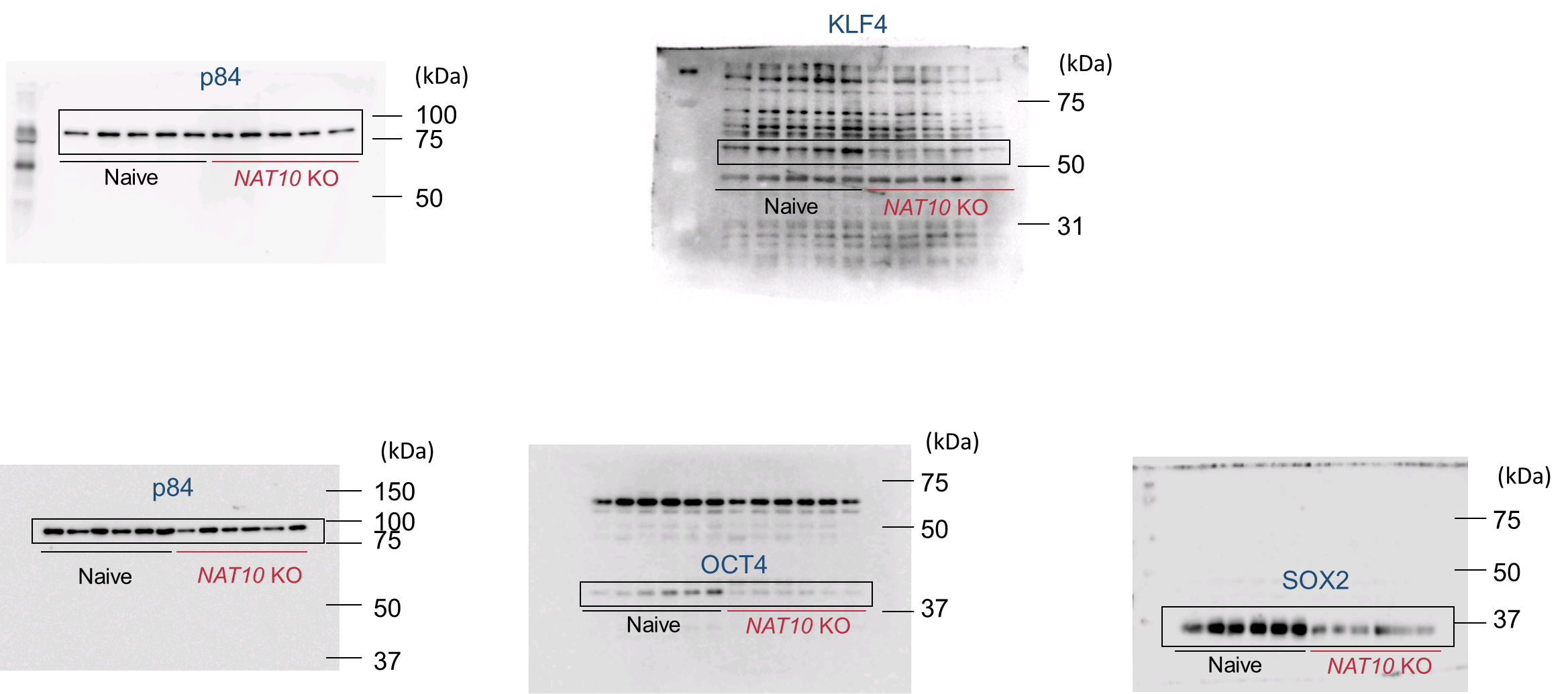
**

**Supplementary Figure S5 Unedited full blots of Figure 2E**

**
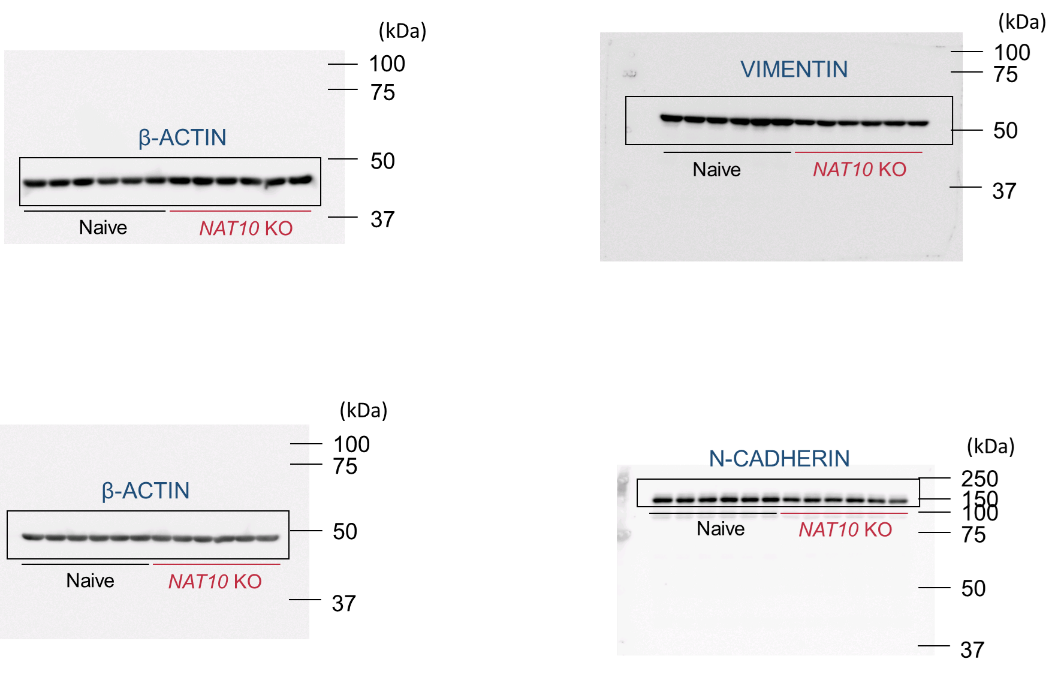
**

**Supplementary Figure S6 Unedited full blots of Figure 2F**

**
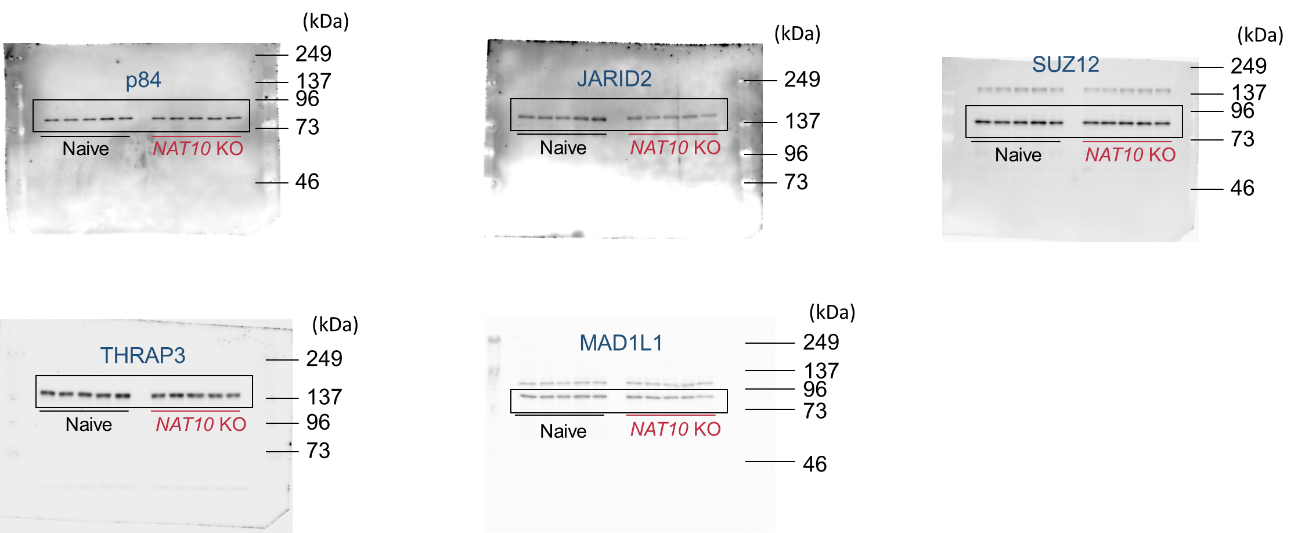
**

**Supplementary Figure S7 Unedited full blots of Figure 3D**

**
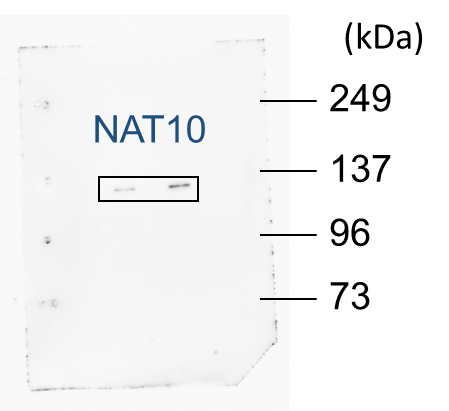
**

**Supplementary Figure S8 Unedited full blots of Figure 4A**

**
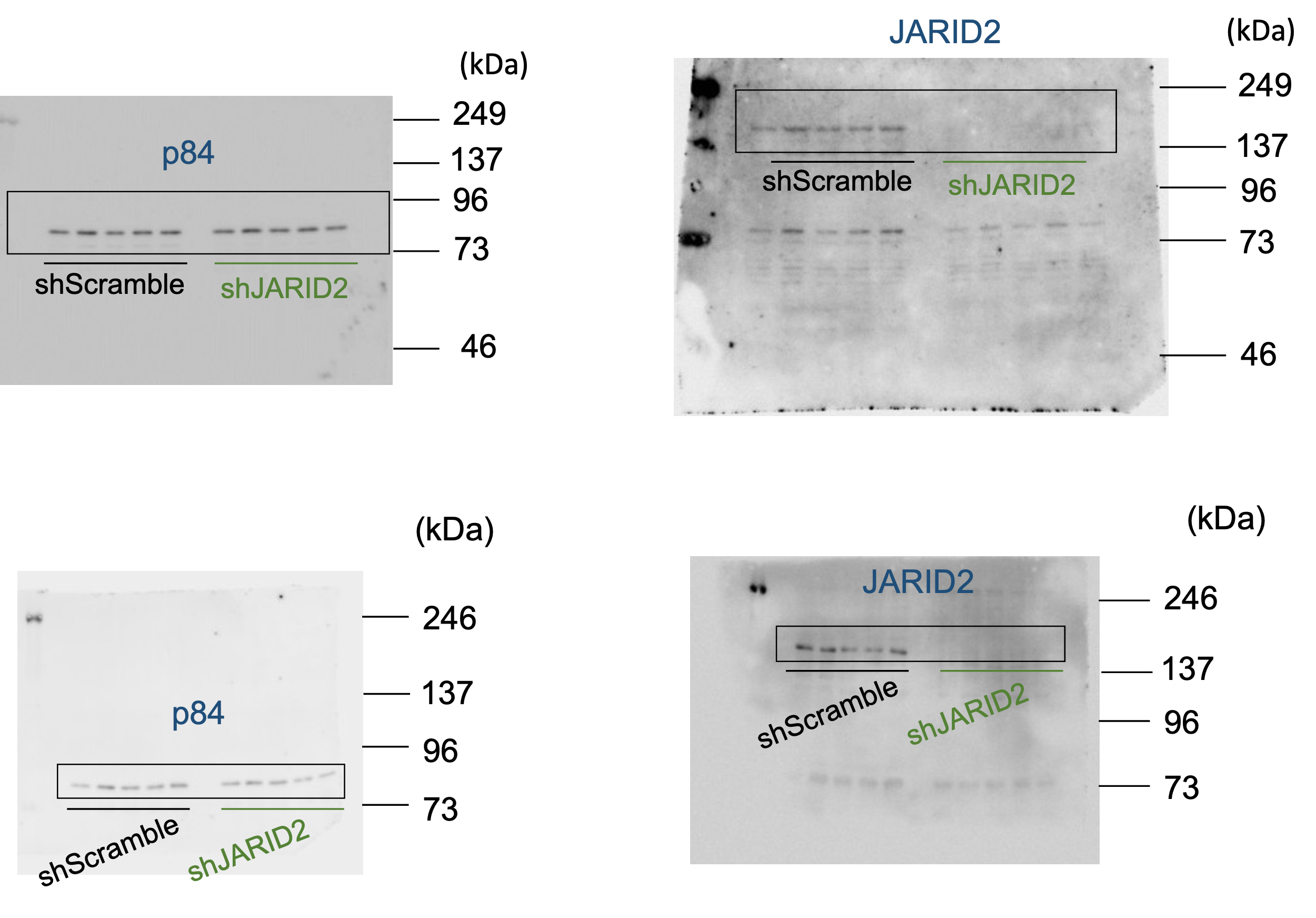
**

**Supplementary Figure S9 Unedited full blots of Figure 5A**

**
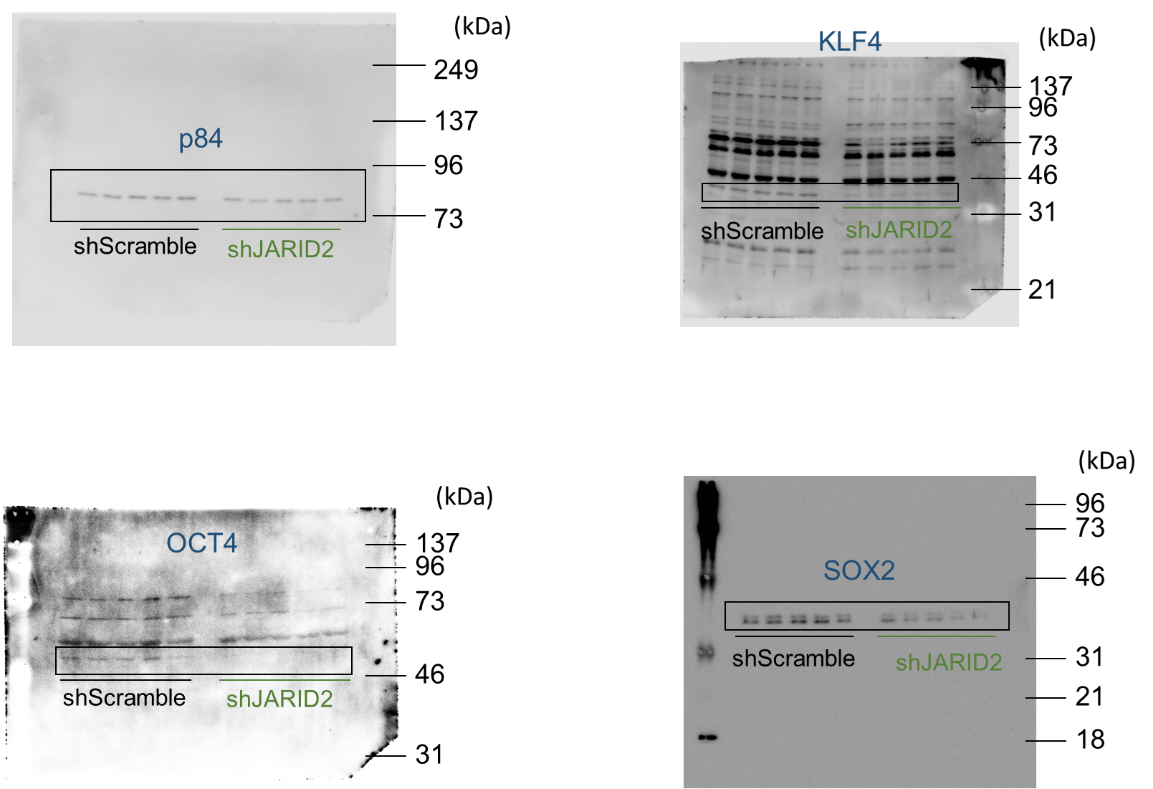
**

**Supplementary Figure S10 Unedited full blots of Figure 5D**

**
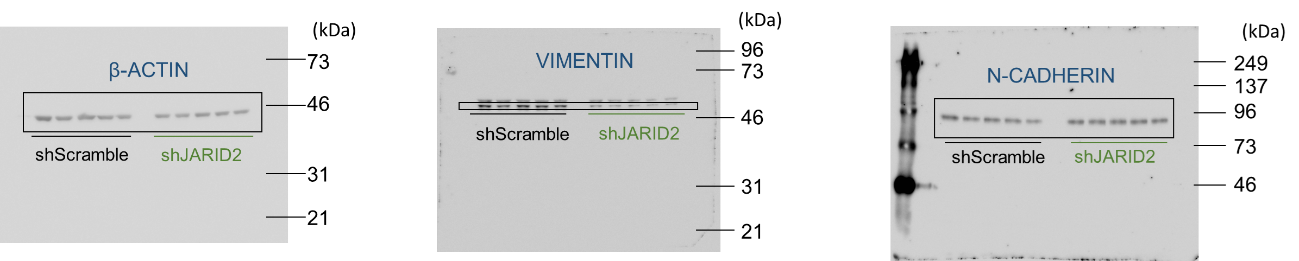
**

**Supplementary Figure S11 Unedited full blots of Figure 5E**
